# The Iron-Sulfur Flavoprotein DsrL as NAD(P)H:Acceptor Oxidoreductase in Oxidative and Reductive Dissimilatory Sulfur Metabolism

**DOI:** 10.3389/fmicb.2020.578209

**Published:** 2020-10-16

**Authors:** Maria Löffler, Kai B. Wallerang, Sofia S. Venceslau, Inês A. C. Pereira, Christiane Dahl

**Affiliations:** ^1^Institut für Mikrobiologie & Biotechnologie, Rheinische Friedrich-Wilhelms-Universität Bonn, Bonn, Germany; ^2^Instituto de Tecnologia Química e Biológica António Xavier, Universidade Nova de Lisboa, Oeiras, Portugal

**Keywords:** dissimilatory sulfate reduction, dissimilatory sulfur oxidation, DsrAB, DsrL, sulfur metabolism, sulfite reductase, NAD(P)H

## Abstract

DsrAB-type dissimilatory sulfite reductase is a key enzyme of microbial sulfur-dependent energy metabolism. Sulfur oxidizers also contain DsrL, which is essential for sulfur oxidation in *Allochromatium vinosum*. This NAD(P)H oxidoreductase acts as physiological partner of oxidative-type rDsrAB. Recent analyses uncovered that DsrL is not confined to sulfur oxidizers but also occurs in (probable) sulfate/sulfur-reducing bacteria. Here, phylogenetic analysis revealed a separation into two major branches, DsrL-1, with two subgroups, and DsrL-2. When present in organisms with reductive-type DsrAB, DsrL is of type 2. In the majority of cases oxidative-type rDsrAB occurs with DsrL-1 but combination with DsrL-2-type enzymes is also observed. Three model DsrL proteins, DsrL-1A and DsrL-1B from the sulfur oxidizers *A. vinosum* and *Chlorobaculum tepidum*, respectively, as well as DsrL-2 from thiosulfate- and sulfur-reducing *Desulfurella amilsii* were kinetically characterized. *Da*DsrL-2 is active with NADP(H) but not with NAD(H) which we relate to a conserved YRR-motif in the substrate-binding domains of all DsrL-2 enzymes. In contrast, *Av*DsrL-1A has a strong preference for NAD(H) and the *Ct*DsrL-1B enzyme is completely inactive with NADP(H). Thus, NAD^+^ as well as NADP^+^ are suitable *in vivo* electron acceptors for rDsrABL-1-catalyzed sulfur oxidation, while NADPH is required as electron donor for sulfite reduction. This observation can be related to the lower redox potential of the NADPH/NADP^+^ than the NADH/NAD^+^ couple under physiological conditions. Organisms with a *rdsrAB* and *dsrL-1* gene combination can be confidently identified as sulfur oxidizers while predictions for organisms with other combinations require much more caution and additional information sources.

## Introduction

Sulfur is a highly reactive element in reduced form and has several stable oxidation states in the range from -2, in sulfide or reduced organic sulfur, up to + 6 in sulfate. The biogeochemical cycle of sulfur on Earth is driven mainly by microbial activity, on one hand by microbial sulfate reduction, on the other by sulfur compound oxidation. Furthermore, sulfur disproportionation is an important process of sulfur-based energy conservation in the absence of oxygen (Finster, 2008). Our understanding of sulfur cycling processes and the biology of microorganisms that catalyze them has improved considerably during recent years (Wasmund et al., 2017; Anantharaman et al., 2018). A wealth of information has become available by molecular biological approaches such as strain-resolution genome reconstruction from metagenomes, single-cell genomics, and other molecular ‘omics’ technologies. Still, significant questions remain regarding the biology of microorganisms and factors that control the turnover of sulfur compounds.

The correct assignment of environmental sequences to the metabolic capabilities of the organisms, requires a thorough understanding of the molecular basis of sulfur-based reductive and oxidative pathways. One important pathway of energy metabolism relying on sulfur is the Dsr-pathway, named after the key enzyme dissimilatory sulfite reductase, DsrAB. This enzyme is not only essential in all dissimilatory sulfate-reducing prokaryotes investigated so far (Venceslau et al., 2014; Rabus et al., 2015) but is also wide-spread in many sulfur oxidizers where it catalyzes the formation of sulfite by oxidation of protein-bound persulfide sulfur in the cytoplasm (Dahl, 2015, 2017, 2020; Tanabe et al., 2019; Löffler et al., 2020). Genes for DsrAB are also present in some microorganisms that are unable to use sulfate including sulfite reducers (Simon and Kroneck, 2013), sulfur-disproportionating bacteria (Finster, 2008; Milucka et al., 2012; Finster et al., 2013), organosulfonate degraders that internally produce sulfite for respiration (*Bilophila wadsworthia*, Laue et al., 2001) and obligate secondary fermenters that apparently have lost the capability for respiring oxidized sulfur compounds (Brauman et al., 1998; Imachi et al., 2006). The genes are commonly used as diagnostic markers in ecological and phylogenetic studies (Wagner et al., 2005; Loy et al., 2009; Müller et al., 2015; Pelikan et al., 2016; Ran et al., 2019) and the ability for a DsrAB-based dissimilatory sulfur metabolism is now predicted in a wide diversity of mesophilic bacterial and archaeal groups including candidate phyla known only based on their genomes (Anantharaman et al., 2018; Hausmann et al., 2018; Zecchin et al., 2018; Thiel et al., 2019). Three main DsrAB protein families are currently discerned (Müller et al., 2015; Pelikan et al., 2016): two reductive types (bacterial and archaeal) and one oxidative bacterial type (reverse-acting DsrAB, rDsrAB). Further branches are represented by the second copies of *dsrAB* in *Moorella* spp. (Loy et al., 2009; Müller et al., 2015) and more recently identified sequences from *Candidatus* Rokubacteria, Verrucomicrobia, and *Candidatus* Hydrothermarchaeota (Anantharaman et al., 2018). Still, predictions on the direction of sulfur metabolism (reductive *vs*. oxidative) for an environmental sequence cannot be solely based on the DsrAB-type as organisms have been described that appear to run an oxidative metabolism with reductive-type DsrAB (Thorup et al., 2017). Predictions therefore usually also take into account co-occurrence of other distinct *dsr* genes, such as those encoding the sulfurtransferase DsrEFH or the iron-sulfur flavoprotein DsrL (Anantharaman et al., 2016; Hausmann et al., 2018). The latter is present in the vast majority of sulfur oxidizer genomes and indeed it has a documented essential function during sulfur oxidation in the purple sulfur bacterium *Allochromatium vinosum* (Lübbe et al., 2006). However, recent sequencing of genomes and metagenomes (Florentino et al., 2017, 2019; Anantharaman et al., 2018; Hausmann et al., 2018) as well as earlier sequencing of large environmental DNA fragments (Mussmann et al., 2005), uncovered the presence of *dsrL-*related sequences also in a number of sulfate-, sulfite-, thiosulfate and/or sulfur-reducing as well as sulfur-disproportionating prokaryotes or in metagenomes encoding reductive-type DsrAB. DsrL forms a complex with rDsrAB in *A. vinosum* and biochemical data point at an *in vivo* function of the complex as a NAD(P)H:sulfite oxidoreductase with the DsrC protein acting as a co-substrate (Löffler et al., 2020). The currently available *dsrL* gene set is largely uncharacterized, thus preventing its use as a marker distinguishing sulfate/sulfite reducers and sulfur oxidizers in newly obtained environmental sequences.

Here, we provide a first step toward a *dsrL* classification system and perform an in depth phylogenetic study of DsrL sequences highlighting the existence of two deep-branching lineages composed of sequences from organism of known physiology as well as from environmental samples. Three representative DsrL proteins are biochemically characterized as recombinant enzymes which enables us to relate sequence characteristics to catalytic properties and thus to function *in vivo.* These analyses provide a framework and guidance for future interpretation of environmental data.

## Materials and Methods

### Bacterial Strains, Plasmids, Primers, and Growth Conditions

Table 1 lists the bacterial strains, and plasmids that were used for this study. *Escherichia coli* strains were grown on lysogeny broth (LB) media under aerobic conditions (Sambrook et al., 1989) at 37°C unless otherwise indicated. Antibiotics were used at the following concentrations (in μg ml^–1^) for all *E. coli* strains, ampicillin 100, kanamycin 50. The pH of buffers and solutions is reported at room temperature.

**TABLE 1:** Strains, plasmids and primers.

**Strains primers or plasmids**	**Relevant genotype, description or sequence**	**References**
**Strains**		
*E. coli* DH5α	*fhuA2* Δ*(argF-lacZ)U169 phoA glnV44* Φ80 Δ*(lacZ)M15 gyrA96 recA1 relA1 endA1 thi-1 hsdR17*	Hanahan, 1983
*E. coli* BL21(DE3) Δ*iscR*	F^–^ *ompT hsdS*_B_ *(r${}_{\text{B}}^{-}$m${}_{\text{B}}^{-}$) gal dcm (DE3) iscR*::kan	Akhtar and Jones, 2008
**Plasmids**		
pET22b	Ap^r^	Novagen
pET22bAvDsrL-CSt	Ap^r^, *NdeI*/*Bam*HI fragment of PCR-amplified *dsrL* in *NdeI*/*Bam*HI of pET22b	Löffler et al., 2020
pET22bCbl.tep DsrL-CSt	Ap^r^, *NdeI*/*Bam*HI fragment of PCR-amplified *dsrL* in *NdeI*/*Bam*HI of pET22b	This work
pET22bD.am DsrL-CSt	Ap^r^, *Nde*I/*Bam*HI fragment of PCR-amplified *dsrL* in *Nde*I/*Bam*HI of pET22b	This work
**Primers**		
LEXf	AGA ACG ATT **CAT ATG** GCG ACT TCC AGC	Lübbe et al., 2006
Rev_*Bam*HI_ CSt_DsrL	GCA TA**G GAT CC**T CAT TTT TCG AAC TGC GGG TGG CTC CAA GCG CTC TCG CCC AGA CCC ATC TTG AT	Löffler et al., 2020
Fwd_*Nde*I_ Cbl.tep_Cst_DsrL	ATT**CATATG**AATGCAGAATCAAACCCGA	This work
Rev_*Bam*HI_ Cbl.tep_Cst_DsrL	ATA**GGATCC**TTATTTTTCGAACTGCGGGTGG CTCCAGCTAGCCAGTCCGTCGCCCATGCCCA	This work
Fwd_*Nde*I_D.am_ CSt_DsrL	ATT**CATATG**GCTGTAGTGAAGGTTAAA	This work
Rev_*Bam*HI_D. am_CSt_DsrL	ATG**GGATCC**CTATTTTTCGAACTGCGGGTGG CTCCAAGCGCTCATTTTTTCTATATAGCCGCA GGGCA	This work

### Recombinant DNA Techniques

Standard techniques for DNA manipulation and cloning were used (Ausubel et al., 1997). Oligonucleotides for cloning were obtained from Eurofins MWG (Ebersberg, Germany).

### Production and Purification of Recombinant DsrL

The green sulfur bacterium *Chlorobaculum tepidum* DSM 12025^T^ contains two different copies of *dsrL*, CT2247 and CT0854 (Frigaard and Dahl, 2009). On the amino acid sequence level the proteins differ at only 4 of 577 positions. None of the respective positions (Ala^286^/Ser^286^, Ile^508^/Val^508^, Asp^550^/Glu^550^ and Thr^552^/Ala^552^) affect co-factor or substrate binding sites such that the properties of the two different DsrL enzymes can be considered essentially identical. Gene CT2247 from *C. tepidum* and the *dsrL* gene from *Desulfurella amilsii* DSM 29984^T^ each with a carboxy-terminal Strep-tag encoding sequence were cloned in pET22b, resulting in plasmids pET22bD.amDsrL-CSt and pET22Cbl.tepDsrL-CSt, and overexpressed in *E. coli* BL21(DE3) Δ*iscR*. One liter batches of LB medium containing 100 mM MOPS buffer pH 7.4, 25 mM glucose and 2 mM iron ammonium citrate as well as 100 μg ml^–1^ ampicillin and 50 μg ml^–1^ kanamycin were inoculated with 5% (v/v) *E. coli* precultures hosting the respective plasmids and cultivated in 2-L flasks at 37°C and 180 rpm until an OD600 of 0.3–0.5 was reached. Cultures were then moved into an anaerobic chamber (Coy Laboratory Products, Grass Lake, United States) containing 98% (v/v) N_2_ and 2% (v/v) H_2_. Cysteine (0.5 mM), sodium fumarate (25 mM) and IPTG (0.4 mM) were added. Cultures were then transferred into completely filled and tightly closed 500-ml bottles, incubated in the absence of oxygen for 65–72 h at 16°C in case of *Da*DsrL-2 production and for 12–16 h at 37°C in case of *Ct*DsrL-1B and harvested by centrifugation (11,000 × *g*, 15 min, 4°C). Cells were resuspended in buffer and lysed by sonication in the anaerobic chamber. After removal of insoluble cell material by centrifugation (16,100 × *g* for 30 min at 4°C), the protein was purified inside the anaerobic chamber by Strep-Tactin affinity chromatography according to the manufacturer’s instructions (IBA Lifesciences, Göttingen, Germany) followed by transfer to 50 mM potassium phosphate buffer, pH 7.0 and concentration to a final volume of about 250 μl via Amicon Ultracel-30K filters (Merck Millipore, Tullagreen, Ireland). The protein was stored under anoxic conditions at −20°C for short time storage and at −70°C for longer time periods. Protein yield was between 15 and 30 mg protein from two liters *E. coli* BL21(DE3) Δ*iscR* culture for both DsrL proteins. *Av*DsrL-1A was produced and purified as described previously (Löffler et al., 2020). Purity of DsrL proteins was assessed by sodium dodecyl sulfate-polyacrylamide gel electrophoresis (SDS-PAGE).

### Protein Techniques and Spectroscopic Analysis

Protein concentrations were determined with the Pierce BCA protein assay kit (Thermo Scientific/Dreieich, Germany). Pure recombinant DsrL proteins were quantified on the basis of their calculated extinction coefficients at 280 nm (67,600, 48,455, and 50,865 M^–1^ cm^–1^ for *Av*DsrL-1A *Ct*DsrL-1B *Da*DsrL-2, respectively). UV-visible absorbance spectroscopy was carried out at 20°C on a Specord 210 UV/Vis spectrophotometer (Analytik Jena/Jena, Germany). The protein samples were prepared in 50 mM potassium phosphate buffer, pH 7.0, and assembled in a quartz glass cuvette (Hellma Analytics/Müllheim, Germany) in the Coy anaerobic chamber. The cuvette was sealed with air-tight septa and titanium(III) citrate (Zehnder and Wuhrmann, 1976) as reductant and potassium ferricyanide as oxidant were added via a gas-tight Hamilton syringe. All spectra were normalized to their absorption at 750 nm.

### Enzyme Assays

All enzyme assays were performed in an anaerobic chamber (98% (v/v) N_2_, 2% (v/v) H_2_) in a final reaction volume of 1 ml. The oxidation/reduction of the electron donor/acceptor was followed with a diode array spectrophotometer (Agilent 8453). Buffers with different pH and different temperatures were tested to determine the optima of the enzymes. NAD(P)H-oxidizing activities of DsrL proteins were measured by following the reduction of 300 μM thiazolyl blue tetrazolium bromide (MTT) at 578 nm (ε = 13 mM^–1^cm^–1^, (Bergmeyer, 1983)]. MTT was dissolved in 75% (v/v) ethanol, 5% (v/v) Triton X-100 and 20% (v/v) H_2_O. 50 mM potassium phosphate buffers at pH 7.0 at 30°C, pH 8.0 at 40°C and pH 6.5 at 45°C were used for *Av*DsrL-1A, *Ct*DsrL-1B and *Da*DsrL-2, respectively. Reactions were started by addition of 0.25 to 1 μg protein. Methylviologen was used as electron donor for NAD(P)^+^ reduction assays which were monitored at 585 nm (ε = 11.8 mM^–1^ cm^–1^). Again varying concentrations of NAD^+^ and NADP^+^ were used. All measurements were performed in triplicates and the median was used for all later calculations. *K*_M_ and *V*_max_ values were calculated and figures were generated with GraphPad Prism 7.

### Bioinformatics, Sequence Alignments and Phylogeny

BLASTP and TBLASTN (NCBI website) were used to find homologs of DsrL from *A. vinosum*. The evolutionary history for DsrL and DsrA was inferred using the Maximum Likelihood method. The analyses involved 143 amino acid sequences for DsrL and 146 DsrA sequences. All ambiguous positions were removed for each sequence pair (pairwise deletion option). There were a total of 852 and 522 positions in the final datasets for DsrL and DsrA, respectively. Evolutionary analyses were conducted in MEGA X (Kumar et al., 2018).

### EPR Spectroscopy and Potentiometric Redox Titration

EPR spectra at X-band were obtained using a Bruker EMX spectrometer equipped with an ESR-900 continuous flow of helium cryostat from Oxford Instruments. Spectra were recorded under the following conditions: microwave frequency, 9.39 GHz; microwave power, 20 mW; modulation frequency, 100 kHz; modulation amplitude, 1 mT; temperature, 15 K. EPR spectra were taken of the as-isolated *Av*DsrL-1A and *Da*DsrL-2, and after sodium dithionite reduction. The EPR-based potentiometric titration was performed inside an anaerobic chamber at 20°C using 115 μM of *Av*DsrL-1A and 50 μM of a mixture of redox mediators in 100 mM MOPS pH 7.5, 5 mM EDTA. The mixture of redox mediators included: methylene blue (+ 11 mV), indigo tetrasulfonate (−30 mV), indigo disulfonate (−110 mV), 2-hydroxy-1,4-naftoquinone (−152 mV), safranin (−280 mV), anthraquinone-2-sulfonate (−225 mV), neutral red (−325 mV), benzyl viologen (−360 mV) and methyl viologen (−446 mV). The potentiometric titration was performed using the as-isolated protein in the reduction direction using buffered sodium dithionite. The reduction potentials were measured with a combined Ag/AgCl electrode calibrated against a saturated quinhydrone solution at pH 7 and referenced to the standard hydrogen electrode. Samples were prepared and transferred to EPR tubes inside the anaerobic chamber, capped and immediately frozen in liquid nitrogen upon removal from the chamber.

### Structural Modeling

Models of DsrL proteins *Av*DsrL-1A, *Ct*DsrL-1B and *Da*DsrL-2 were separately predicted with I-Tasser (Roy et al., 2010; Yang and Zhang, 2015) for the main protein bodies (amino acid sequence alignment positions 1-627) and for the carboxy-terminal part consisting of the linker and ferredoxin domains. Predicted structures for the main protein bodies, the carboxy-terminal parts and cofactors predicted by I-TASSER were joined using the UCSF Chimera package (Pettersen et al., 2004).

## Results

### Identification of *DsrL* in Organisms/Metagenomes With/Related to Sulfur-Based Energy Metabolism

**FIGURE 1:**
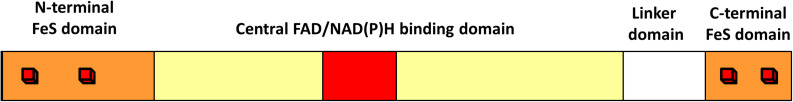
Common structure of DsrL proteins. All DsrL proteins consist of an amino-terminal ferredoxin domain (orange), a central domain (the NAD(P)-binding domain depicted in red is embedded in the FAD-binding domain highlighted in yellow), a linker domain and a carboxy-terminal ferredoxin domain (orange). Red cubes illustrate [4Fe-4S] clusters.

A double-tracked approach was performed to reveal the distribution of *dsrL* sequences. In the first step, all completed and yet unfinished publicly accessible genome sequences were screened for the presence of *dsrL* by BLAST (Altschul et al., 1990). DsrL belongs to a large protein family, the FAD and FeS cluster-containing pyridine nucleotide:disulfide oxidoreductases (Dahl et al., 2005). Accordingly, sequence-related but functionally clearly different proteins such as the small subunits of glutamate synthases (GltD) or the structurally most closely DsrL-related Nfn proteins appear as frequent results in such searches. All results were therefore manually curated and only those sequences that fulfilled the following criteria were further considered: [1] The gene stems from an organism containing *dsrAB* genes and the putative *dsrL* gene is complete. [2] The encoded protein contains all DsrL-specific domains in the correct order, i.e., an N-terminal two-[4Fe-4S]-ferredoxin domain, one Rossmann-type nucleotide-binding domain for FAD with an embedded second Rossmann-type nucleotide-binding domain for NAD(P) and a second two-[4Fe-4S]-ferredoxin domain situated at the carboxy-terminus, that is connected to the N-terminal body of the protein via a linker with a length of ∼100 amino acids (Figure 1; Löffler et al., 2020). [3] The gene is not immediately linked with genes annotated as subunits of pyruvate/ketoisovalerate: ferredoxin oxidoreductases. These were also excluded from further analyses.

**FIGURE 2:**
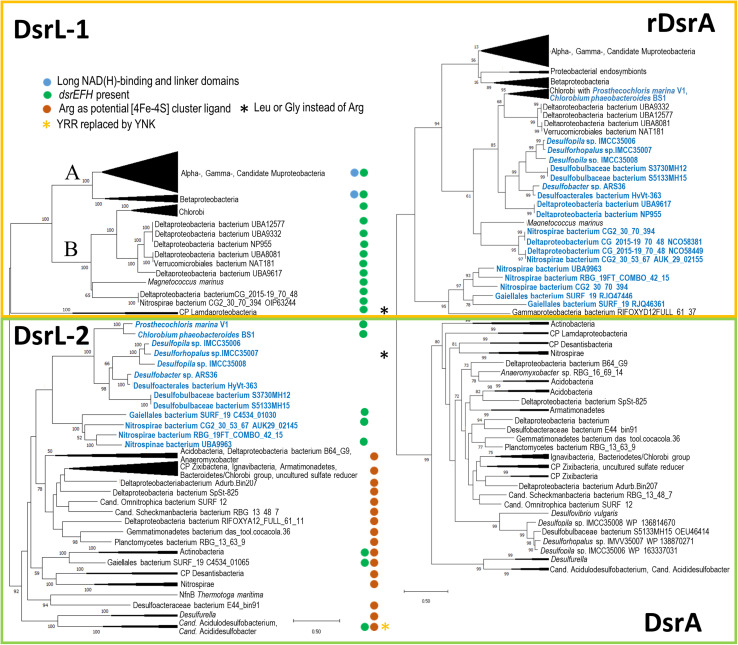
Comparison of DsrL **(A)** and DsrA **(B)** trees. Trees were constructed by using the Maximum Likelihood method with 1000 bootstrap resamplings. First, the best amino acid substitution models were calculated in MEGA X (Kumar et al., 2018). For DsrA as well as for DsrL, the Le_Gascuel_2008 model (Le and Gascuel, 2008) had the lowest BIC (Bayesian Information Criterion) score and was considered to describe the substitution pattern the best. Initial tree(s) for the heuristic search were obtained automatically by applying Neighbor-Join and BioNJ algorithms to a matrix of pairwise distances estimated using the JTT model, and then selecting the topology with superior log likelihood value. A discrete Gamma distribution was used to model evolutionary rate differences among sites (5 categories [+ *G*, parameters = 1.1935 and 0.8872 for DsrL and DsrA, respectively)]. The rate variation model allowed for some sites to be evolutionarily invariable ([ + *I*], 2.11% and 5.17% sites for DsrL and DsrA, respectively). Bootstrap values exceeding 50% are given at branching points. For DsrL and DsrA, the optimal trees with the highest log likelihood –76420.42 and –39346.85, respectively, are shown. The trees are drawn to scale, with branch lengths measured in the number of substitutions per site. Neighbor-joining phylogenies were also calculated and yielded essentially the same results. Complete phylogenetic trees with bootstrap values are available as Supplementary Data Files 1 and 2. It should be noted that some DsrL sequences reported earlier in Actinobacteria bacterium GWC2_53_9, Deltaproteobacteria bacteria RIFOXYA12_FULL_58_15 and RIFOXYB12_FULL_58_9, *Candidatus* Rokubacteria bacterium RIFCSPLOWO2_02_FULL_68_19, and *Nitrospinae* bacterium RIFCSPLOWO2_01_FULL_39_10 (Anantharaman et al., 2018) could not be integrated into phylogenetic tree construction because of too many ambiguous residues. A yellow box encloses all oxidative-type DsrA (rDsrA) proteins as well as all DsrL-1 type proteins. A green box features all bacterial reductive-type DsrA and DsrL-2-type proteins. Lineages in blue contain oxidative type DsrA and a DsrL protein of type DsrL-2. All DsrL sequences in group DsrL-2 feature a YRR motif indicative of preference for NADP(H) over NAD(H). *Candidatus* Acidulodesulfobacterium and *Candidatus* Acididesulfobacter species are the only exceptions. Here, YRR is replaced by YNK (yellow asterisk). Blue dots indicate the presence of long substrate binding and linker domains. Brown dots highlight DsrL proteins with an arginine instead of cysteine as potential [4Fe-4S] cluster ligand in the N-terminal ferredoxin domain (cf. Figure 3). Organisms highlighted with a black asterisk exhibit leucine or glycine at this position. Green dots indicate organisms containing genes for the sulfurtransferase DsrEFH.

Our searches yielded many DsrL sequences from established DsrAB-containing sulfur-oxidizing bacteria belonging to the Alpha-, Beta- and Gammaproteobacteria as well as the green sulfur bacteria (phylum Chlorobi). We therefore restricted our analyses to well-studied representatives of these groups (Figure 2A and Supplementary Table 1). The DsrAB-containing organism group implicated in sulfur oxidation has recently been widened by three additional lineages, Nitrospirae, Nitrospinae and *Candidatus* Muproteobacteria (Anantharaman et al., 2018) and indeed, DsrL-encoding genes are also present in representatives of these groups. In addition, *dsrL* genes were unambiguously identified in some representatives of the Lambdaproteobacteria, Acidobacteria, Actinobacteria, Chloroflexi, Armatimonadetes, Gemmatimonadetes, Planctomycetes, Ignavibacteria, Verrucomicrobia and the candidate phyla Schekmanbacteria, Desantisbacteria and Zixibacteria (Figure 2A and Supplementary Table 1). While previous predictions regarding dissimilatory sulfur metabolism were inconclusive for representatives of the Actinobacteria, Acidobacteria, Lambdaproteobacteria and Verrucomicrobia (sulfate/sulfite reduction or sulfur oxidation or both), the ability to reduce sulfate/sulfite was suggested for the other taxa (Anantharaman et al., 2018). The presence of a *dsrL* gene in *Candidatus* Omnitrophica bacterium isolate SURF_12, a putative sulfate/sulfur reducer, was somewhat surprising because it remained undetected in an earlier survey (Momper et al., 2017). DsrL is neither present in archaeal sulfate reducers nor was it uncovered in the candidate phyla Falkowbacteria, Hydrothermarchaeota, Riflebacteria and Rokubacteria, all of which have been implicated in DsrAB-based sulfate reduction (Anantharaman et al., 2018).

With regard to the presence of *dsrL*, the Deltaproteobacteria constitute an interesting group. In complete agreement with earlier surveys, DsrL is not encoded in any of the classical deltaproteobacterial sulfate reducers, e.g., the genus *Desulfovibrio*. In addition, DsrL is neither present in any of the filamentous cable bacteria, e.g., from the candidate genera Electrothrix and Electronema (Risgaard-Petersen et al., 2015; Trojan et al., 2016; Kjeldsen et al., 2019; Müller et al., 2019) nor in sulfide-oxidizing *Desulfurivibrio* species that cannot be distinguished from canonical sulfate-reducing bacteria using gene synteny or other genomic features (Thorup et al., 2017). On the other hand, the presence of *dsrL* in *Desulfurella amilsii*, an organism described as sulfur and thiosulfate reducer with the additional capacity for sulfur disproportionation, and in *Candidatus* Acididesulfobacter and *Candidatus* Acidulodesulfobacterium species that have been proposed to be capable of both sulfate reduction as well as sulfide oxidation has been noted and discussed earlier (Florentino et al., 2019; Tan et al., 2019), while its occurrence in a number of unclassified deltaproteobacterial metagenomes and metagenomes assigned to the families Desulfobacteraceae and Desulfobulbaceae has not attracted attention so far.

### Correlation of DsrL and DsrA Phylogenies

Based solely on the established or predicted physiology of the source organisms, DsrL appeared to occur in sulfur oxidizers as well as in sulfate/sulfite reducers, sulfur disproportionating organisms and bacteria proposed to be capable of switching between these metabolisms. To further investigate the metabolic diversity of microorganisms that contain DsrL, we performed phylogenetic analysis of sequences listed in Supplementary Table 1 and correlated it with a tree derived for DsrA sequences from the studied organisms/metagenomes.

Phylogenetic analysis of DsrL proteins indicated two main branches, termed DsrL-1 and DsrL-2, that coincided with the absence *vs.* presence of a characteristic YRR motif in group DsrL-1 and DsrL-2 sequences, respectively (Figure 2A). The function of the YRR motif in binding of the nicotinamide adenine dinucleotide (phosphate) cofactor is discussed below. The first large group, DsrL-1 is subdivided into two types (DsrL-1A and DsrL-1B). DsrL-1A sequences almost exclusively stem from established or suggested sulfur oxidizers. Among group DsrL-1B this holds true for DsrL from green sulfur bacteria as well as from *Magnetococcus marinus* while the physiology of the DsrL-1B-containing unclassified Deltaproteobacteria, Verrucomicrobiales bacterium NAT181 and Nitrospira bacterium CG2_30_70_394 is unclear.

The second large DsrL group (DsrL-2) comprises proteins from two green sulfur bacteria, *Chlorobium phaeobacteroides* and *Prosthecochloris marina* V1 that are established sulfur oxidizers (Imhoff, 2014; Bryantseva et al., 2019). These sequence are located on the same major branch as DsrL-2 proteins assigned to members of the Nitrospirae, one Nitrospinae bacterium, Actinobacteria (Gaiellales bacterium SURF_19) and several Deltaproteobacteria including strains of the genera *Desulfopila* and *Desulforhopalus*. In none of the cases anything is known about the physiology of the organisms except that other species of the genera *Desulfopila* and *Desulforhopalus* have been characterized as sulfate reducers (Isaksen and Teske, 1996; Suzuki et al., 2007). Other branches within group DsrL-2 feature sequences stemming from organisms of very different taxonomic affiliation ranging from candidate phyla Zixibacteria, Desantisbacteria and Schekmanbacteria to members of the Armatimonadetes and Acidobacteria as well as a whole range of Deltaproteobacteria including *Desulfurella* species (Figure 2A).

As expected, the DsrA tree yielded almost the same results as those reported for trees of concatenated DsrAB sequences (Müller et al., 2015; Anantharaman et al., 2018; Tan et al., 2019). We identified two major groups representing oxidative-type reverse Dsr (rDsrA) and reductive bacterial-type DsrA (Figure 2B). Oxidative-type rDsrA included all sequences from Alpha-, Beta-, Gamma- and *Candidatus* Muproteobacteria as well as those from green sulfur bacteria. In addition, several Nitrospirae bacterium strains featured oxidative-type rDsrA including RBG_19FT_COMBO_42_15 that had been grouped exactly the same by Anantharaman et al. (2018). Furthermore, oxidative-type rDsrA was found in several Deltaproteobacteria including metagenomes assigned to *Desulfopila* and *Desulforhopalus.* In agreement with Anantharaman et al. (2018), the reductive-type DsrA branch included Lambdaproteobacteria and the candidate phyla Zixibacteria, Schekmanbacteria and Desantisbacteria as well DsrA from Ignavibacteria, Actinobacteria and Armatimonadetes, albeit deeper branching points did not exactly match in all cases.

Comparing the DsrL and DsrA trees provided in Figure 2 proved revealing. In fact, the presence of DsrL-1 correlates with the presence of oxidative-type rDsrA without exception, i.e., all DsrL-1 sequences co-exist in the same organism with rDsrA. Furthermore, all organisms/metagenomes with reductive bacterial-type DsrA contain DsrL of type DsrL-2. Interestingly, the situation is not so straight forward for a group of oxidative-type rDsrA-containing Deltaproteobacteria and Nitrospirae, Nitrospinae bacterium UB9963, a member of the Actinobacteria (Gaiellales bacterium SURF19) and for two Chlorobi, *Prosthecochloris marina* V1 and *Chlorobium phaeobacteroides* BS1 (Figure 2). DsrL encoded in these genomes is of type DsrL-2. The only organisms in this group with established physiology are the two green sulfur bacteria, that clearly thrive as sulfur compound oxidizers (Imhoff and Thiel, 2010; Bryantseva et al., 2019).

### Prevalence of *DsrEFH* in *DsrL*-Containing Organisms

The protein DsrEFH is essential for sulfur oxidation in the purple sulfur bacterium *A. vinosum*. This sulfur trafficking enzyme mediates transfer of sulfur atoms delivered by yet another sulfurtransferase, TusA, to the sulfur carrier protein DsrC, which then provides rDsrAB with oxidizable sulfur (Dahl et al., 2008; Stockdreher et al., 2012, 2014; Tanabe et al., 2019; Dahl, 2020; Löffler et al., 2020). Over the last years, we have repeatedly suggested that the *dsrEFH* genes are unique to sulfur oxidizers and absent from sulfate/sulfite reducers and sulfur disproportionating organisms and may therefore be indicators for sulfur metabolism operated in the oxidative direction (Sander et al., 2006; Stockdreher et al., 2012; Venceslau et al., 2014). However, this concept has been seriously challenged by the presence of the genes in organisms that are unlikely to be sulfur oxidizers, i.e., in *Candidatus* Rokubacteria (Anantharaman et al., 2018). In addition, other organisms containing *dsrEFH* such as *Candidatus* Acidulodesulfobacterales species have been suggested as being able to do both reduce sulfate and oxidize sulfide depending on environmental conditions (Tan et al., 2019). Correspondingly, the latter also harbor the genetic potential to oxidize thiosulfate (Tan et al., 2019). It should also be emphasized here, that a lack of DsrEFH does not necessarily lead to an inability to oxidize sulfur as has been shown for *D. alkaliphilus*, an organism that performs sulfide oxidation with the *dsr* gene set of a typical sulfate reducer without DsrEFH (Thorup et al., 2017).

Still, we considered a survey of *dsrL*-containing organisms for the presence of *dsrEFH* genes informative and, indeed, a strict connection between the occurrence of oxidative-type rDsrA, DsrL-1 and the presence of DsrEFH is observed (Figure 2 and Supplementary Table 1) which strongly substantiates the combined occurrence of the corresponding genes as a reliable predictor for dissimilatory sulfur oxidation. Only a few organisms/metagenomes encoding reductive-type DsrA and a DsrL-2 type protein feature DsrEFH. Notable exceptions with DsrEFH are representatives of the Actinobacteria and three species of the order *Candidatus* Acidulodesulfobacterales as has been pointed out previously (Tan et al., 2019). Just as proposed for *Candidatus* Acidulodesulfobacterales these organisms could in principle be capable of both sulfur oxidation and sulfate reduction albeit this has never been experimentally proven for any organism so far. All of the remaining organisms contain oxidative-type rDsrAB and their DsrL-2 proteins are related to that from the green sulfur bacterium *P. marina* V1. In this group, the presence of *dsrEFH* gene does not follow an obvious rule. While the two sulfur-oxidizing green sulfur bacteria as well as representatives of the Actinobacteria, Nitrospirae and Nitrospinae have DsrEFH, the corresponding genes are not present in the Deltaproteobacteria clustering here (Figure 2).

### Special Gene Arrangements

Some organisms/metagenomes feature remarkable gene arrangements that may contribute to answering the question of which mode of sulfur metabolism is operated. In Myxococcales bacterium SURF_8, oxidative-type DsrA and DsrL-1A are encoded in a *dsrABEFHCMKL_1_L_2_JOP* cluster, pointing at sulfur oxidation. DsrL from *A. vinosum* has been characterized as a functional homodimer (Löffler et al., 2020) and it is thus conceivable that a DsrL_1_L_2_ heterodimer is formed in strain SURF_8. The cluster is preceded by a gene annotated as *tauD* that is transcribed in the opposite direction. Similar gene arrangements have been noted by Lenk et al. (2012), who termed the *tauD*-like gene *dsrQ* and found it upstream of nearly all the fosmid clones from the *Roseobacter* clade they sequenced and also in most (facultatively) aerobic chemotrophic sulfur oxidizers they analyzed in their study. In our *dsrL*-containing organisms, the co-occurrence of *tauD/dsrQ* and *dsr* genes is not restricted to strain SURF_8 but also found in Deltaproteobacteria bacterium isolates UBA12577, UBA9332 and UBA8081 and in Verrucomicrobiales bacterium isolate NAT181 in a *dsrNCABL-hyp-dsrQ-dsrEFH* cluster. It has been speculated that the TauD/DsrQ protein catalyzes the release of sulfite during oxygenolytic breakdown of intracellular or ambient sulfonates. Possibly, sulfite is then disproportionated to sulfate and sulfide as has been found for cysteate and isethionate (Denger et al., 1999). A cluster very similar to that of the *dsr-tauD/dsrQ* combination is present in Deltaproteobacteria bacterium isolates UBA9617 and NP955 but with a replacement of *tauD* by a *tusA*-like gene. TusA is a well-established sulfurtransferase involved in the delivery of sulfur to DsrEFH in the sulfur oxidizer *A. vinosum* (Stockdreher et al., 2014; Tanabe et al., 2019)

Apart from Myxococcales bacterium SURF_8, we found one further organisms with two *dsrL* genes. The genome of Nitrospirae bacterium CG2_30_53_67 not only contains two *dsrL* but also two copies of *dsrAB.* The first DsrL protein resides in the second major DsrL-2 group (Figure 2) and the gene is located in immediate vicinity of the genes for reductive-type sulfite reductase in an *dsrABDL-2* arrangement as has been depicted previously by others (Tan et al., 2019). The second protein is found among those resembling *P. marina* V1 DsrL-2 (Figure 2) and is situated in an *rdsrABL-2* arrangement encoding oxidative-type rDsrAB. This gene cluster went unnoticed so far. A *dsrTMKJOPCEFH* cluster is located elsewhere in the genome (Tan et al., 2019). It is possible that Nitrospirae bacterium CG2_30_53_67 contains one *dsrABL* set specifically adapted to sulfur oxidation and the other specialized for sulfite reduction. Gailellales bacterium SURF19 even contains three different *dsrAB* sets and three different *dsrL* genes. Both DsrA proteins encoded in the *dsrNMCKJOPABDL-2-hyp(PAS/Pac sensor domain protein)-dsrEFHABL-2* cluster are of the oxidative type, while the two DsrL enzymes fall into the two different major DsrL-2 groups (Figure 2). The third DsrA clusters together with reductive bacterial type DsrA from other Actinobacteria and is encoded in a *dsrABL* set. The *dsrL* gene is incomplete and the encoded protein could not be phylogenetically analyzed. Still, our observations suggest that Gailellales bacterium SURF19 may well be able to switch between oxidative and reductive dissimilatory sulfur metabolism.

*Desulfopila* sp. strain IMC35006 and IMC35008, *Desulforhopalus* sp. IMC35007 and Desulfobulbaceae bacterium S5133MH15 make up interesting cases because they each contain two copies of the *dsrAB* genes, one of which is of the oxidative-type (*rdsrABL-2* arrangement) and the other of the bacterial reductive type (*dsrABD* arrangement, except of strain S5133MH15 in which the contig ends with *dsrB*), again suggestive of the capability to run sulfur metabolism in both directions. Genes *dsrC* and *dsrMKJOP* reside elsewhere in the genomes that lack *dsrEFH* genes.

### Sequence Characteristics of the Different DsrL Types

Given our interest in possible prediction of metabolic features from (meta)genomic features and linking these with biochemical data, we went ahead and identified sequence characteristics of the different DsrL types and related these to catalytic properties.

**FIGURE 3:**
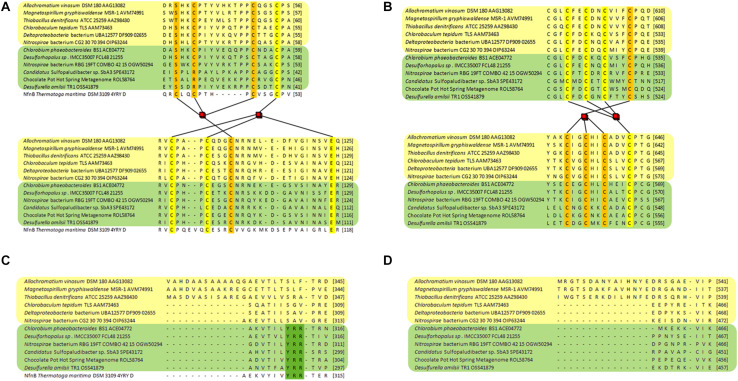
Partial sequence alignments of DsrL proteins. DsrL-1 group proteins are highlighted in yellow and proteins belonging to group DsrL-2 are highlighted in green. DsrL from *Allochromatium vinosum*, *Magnetospirillum gryphiswaldense* and *Thiobacillus denitrificans* falls within the DsrL-1A branch, while the proteins from *Chlorobaculum tepidum*, Deltaproteobacteria bacterium UBA12577 and Nitrospirae bacterium CG2_20_70_394 are representatives of the DsrL-1B group. The DsrL-2 proteins from *Chlorobium phaeobacteroides* BS1, *Desulforhopalus* sp. IMCC35007 and Nitrospirae bacterium RBG_19FT_COMBO_42_15 stem from organisms with oxidative-type rDsrA, while the DsrL-2 from *Candidatus* Sulfopaludibacter sp. SbA3, the Chocolate Pot Hot Spring Metagenome and *Desulfurella amilsii* are encoded together with reductive bacterial-type DsrA. **(A)** The amino-terminal region binding two [4Fe-4S] clusters is shown. Predicted iron ligands are highlighted in orange and yellow for the distal and the proximal cluster, respectively. **(B)** Iron-liganding cysteines in the two [4Fe-4S] cluster-binding carboxy-terminal ferredoxin domain are marked in yellow and orange for each of the clusters. **(C)** The NAD(P)-binding domain of the different DsrL proteins is compared revealing part of an extension for the DsrL-1A proteins and the presence of the YRR motif indicative of interaction with NADP in DsrL-2-type enzymes. **(D)** Part of the linker domain connecting the major protein body with the carboxy-terminal ferredoxin domain showing an extension for DsrL-1A-type enzymes. The respective regions in the structurally characterized and DsrL-related protein NfnB from *Thermotoga maritima* (Demmer et al., 2015) are shown for comparison in panels **A**,**C**.

**FIGURE 4:**
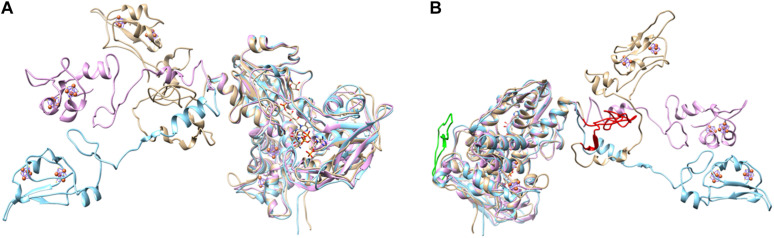
Two different views on the overlayed modeled structures of *Av*DsrL-1A (beige), *Ct*DsrL-1B (light blue) and *Da*DsrL-2 (violet). For clarity, FAD, NAD and [4Fe-4S] clusters in the amino-terminal ferredoxin-domain are only shown for the protein from *A. vinosum*. All these prosthetic groups/substrates were modeled at the equivalent positions in *Ct*DsrL-1B and *Da*DsrL-2. **(A)** The view shown here illustrates the different possible positions for the carboxy-terminal ferredoxin domains shown in the left part of the figure. **(B)** The view provided here highlights in red and green, respectively, the extensions present in the substrate-binding domain (amino acids 302–333) and the linker domain (amino acids 485-532) of *Av*DsrL-1A.

Analysis of DsrL sequence alignments reveals several diagnostic differences between the different groups. [1] In the N-terminal ferredoxin domain most of the cysteines binding the two [4Fe-4S] clusters in the related NfnB protein from *Thermotoga maritima* (Demmer et al., 2015) are conserved in the DsrL proteins. This applies to Cys^47^ and Cys^100^ (NfnB numbering) coordinating the distal cluster and Cys^51^, Cys^90^, Cys^96^ and Glu^117^ binding the proximal cluster (Figure 3A). Notably, Cys^39^, the third ligand of the distal NfnB cluster is replaced by either serine or threonine in all DsrL sequences (Figure 3A). Both residues can serve as alternative iron ligands (Bak and Elliott, 2014; McLaughlin et al., 2016). The fourth ligand of the distal cluster (corresponding to Cys^42^ in NfnB) is a cysteine in DsrL-1 and DsrL-2 stemming from organisms with oxidative type rDsrA but replaced by arginine in the other DsrL-2 proteins (Figures 2, 3A). Arginine can also serve as an iron ligand and has been associated with a reduction of cluster redox potential (Bak and Elliott, 2014). In summary, the vast majority of DsrL amino-terminal domains have the theoretical capacity for binding two [4Fe-4S] clusters. The only exceptions to this rule are the two Lambdaproteobacteria analyzed and *Desulfopila* sp. IMCC3006 which feature leucine and glycine, respectively, at the position corresponding to Cys^42^ in NfnB. In the carboxy-terminal ferredoxin domains of DsrL proteins all FeS-cluster ligands are cysteines that are strictly conserved (Figure 3B). [2] All proteins falling into group DsrL-1A exhibit a significantly longer substrate binding domain due to a ∼32 amino-acid insertion in the NAD(P)-binding site. This insertion appears to form an additional loop as evident by comparison of the modeled structures for three typical DsrL proteins, DsrL-1A from *Allochromatium vinosum* [*Av*DsrL-1A (Löffler et al., 2020)], DsrL-1B from *Chlorobaculum tepidum* (*Cb*DsrL-1B) and DsrL-2 from *Desulfurella amilsii* (*Da*DsrL2) (Figure 4). [3] All proteins in the DsrL-1A group exhibit linker domains that are ∼30 amino acids longer than those in the other groups (Figure 3D). The linker domains appear to connect the carboxy-terminal ferredoxin with the main protein body (Figure 4). The linkers are predicted to adopt very flexible structures enabling movement of the linker as obvious by the different positions of the carboxy-terminal regions in the three superimposed DsrL models. A longer linker region would potentially allow more freedom for positioning of the ferredoxin domain (Figure 4B).

### Functional Characteristics of DsrL-1A, DsrL-1B, and DsrL-2 Proteins

**FIGURE 5:**
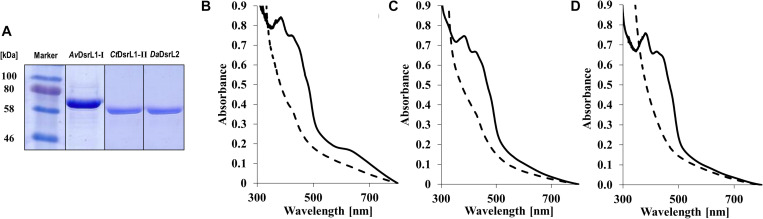
**(A)** SDS-PAGE of DsrL proteins after streptactin-based affinity purification. Lane 1, 30 μg *Av*DsrL1-I, lane 2, 25 μg *Cb*DsrL1-II, lane 3, 25 μg *Da*DsrL2. **(B–D)** UV vis spectra of recombinant DsrL proteins. **(B)** *Av*DsrL-1A, **(C)** *Ct*DsrL-1B, **(D)** *Da*DsrL-2. Spectra were normalized to 40 μM. Solid lines, spectra of proteins as isolated. Dashed lines, proteins reduced by addition of 1–4 times molar excess of titanium(III) citrate.

The conspicuous occurrence of DsrL-1 type proteins exclusively in sulfur oxidizers and the association of DsrL proteins of the second type (DsrL-2) with a number of established or predicted sulfite/sulfate reducers suggested possible functional adaptation of the various DsrL enzymes. In order to challenge this notion on an experimental basis, we produced and characterized recombinant DsrL proteins belonging to three distinct groups and stemming from organisms with well-established physiology: DsrL-1A from the phototrophic sulfur oxidizer *A. vinosum*, DsrL-1B from the green sulfur bacterium *C. tepidum* and DsrL-2 from *D. amilsii*. As already described for *Av*DsrL-1A (Löffler et al., 2020), all three proteins were produced with carboxy-terminal Strep-tags in *E. coli* BL21(DE3) Δ*iscR* grown anaerobically on fumarate (Kuchenreuther et al., 2010). The Δ*iscR* strain is engineered for improved synthesis of iron–sulfur proteins by the removal of the gene for IscR, a transcriptional negative regulator of the *isc* (iron–sulfur cluster biosynthesis) operon (Akhtar and Jones, 2008). All three proteins were obtained in electrophoretically pure form (Figure 5A). Just as the protein from *A. vinosum*, the enzymes from *C. tepidum* and *D. amilsii* exhibited a brown color and the typical UV-vis spectroscopic characteristics of iron-sulfur flavoproteins (Figures 5B–D). The flavin cofactor in *Av*DsrL-1A has previously been identified as flavin adenine dinucleotide and quantitative analyses of FAD as well as iron and sulfur were fully in line with one FAD and four [4Fe-4S] clusters per DsrL monomer as predicted from the sequence (Löffler et al., 2020). Spectra in the visible range of the three recombinant DsrL proteins normalized to 40 μM (Figures 5B–D) revealed a similar albeit somewhat lower degree of cofactor loading for the enzymes from *C. tepidum* and *D. amilsii*.

**FIGURE 6:**
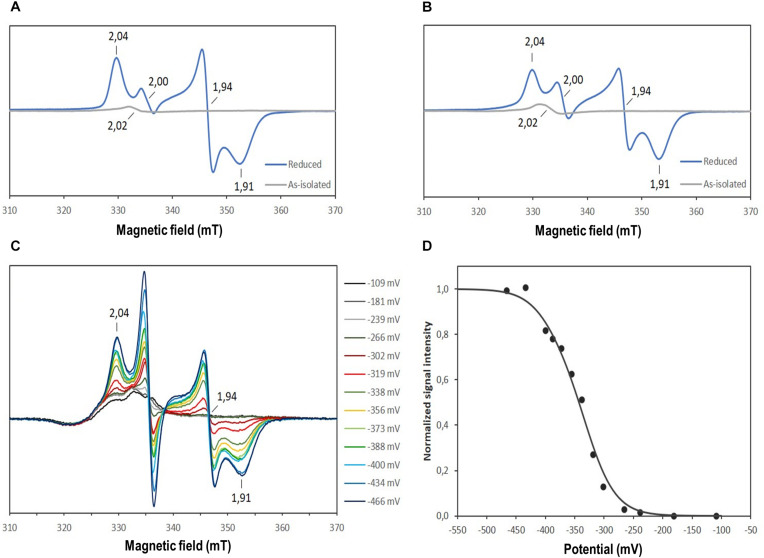
EPR spectra of the as-isolated and reduced *Av*DsrL-1A **(A)** and *Da*DsrL-2 **(B)**, EPR spectra taken during the redox titration of *Av*DsrL-1A **(C)** and redox titration curve following the *g* value of 1.94 **(D)**. The best fit to the experimental data was achieved by assuming reduction of four Fe-S centers in a 3:1 ratio with a *E*_m_ value of –330 mV and –390 mV, respectively.

The nature of the iron-sulfur clusters in DsrL was studied by electron paramagnetic resonance spectroscopy. The EPR spectra of as-isolated *Av*DsrL-1A and *Da*DsrL-2 showed a very weak isotropic signal centered at *g* = 2.02 that suggests the presence of a [3Fe-4S]^+^ cluster, which is paramagnetic in the oxidized state. Both reduced *Av*DsrL-1A reduced *Da*DsrL-2 exhibited similar rhombic signals characteristic of [4Fe-4S]^+^ clusters (Figures 6A,6B). Integration of the relative intensity of the [4Fe-4S]^+^ signal *vs*. the [3Fe-4S]^+^ signals yielded a value of 22 times higher for *Av*DsrL-1A and 19 times higher for *Da*DsrL-2, which indicates that the intensity of the [3Fe-4S]^+^ signal in the as-isolated proteins corresponds to only 0.18 and 0.21 of a center, respectively. This suggests that in both cases the signal from the [3Fe-4S]^+/0^ center probably results from some small degradation of the [4Fe-4S]^2+/+^ clusters.

**FIGURE 7:**
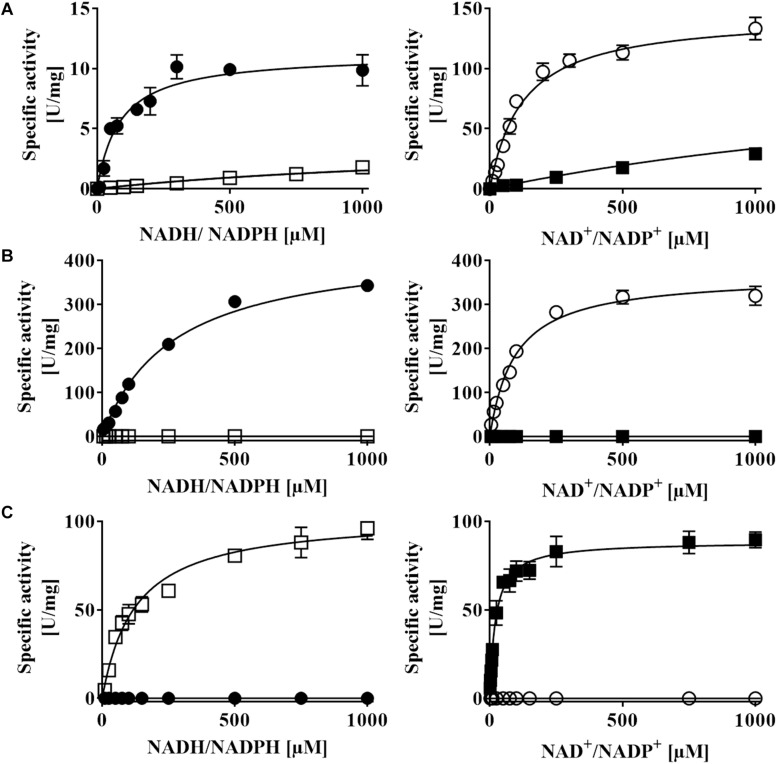
NAD(P)H:acceptor oxidoreductase activity of recombinant **(A)** *Av*DsrL-1A, **(B)** *Ct*DsrL-1B, and **(C)** *Da*DsrL-2. Activities were determined under anoxic conditions applying optimal pH and temperature for each enzyme. MTT was used as artificial electron acceptor in the NADH/NADPH oxidizing direction and reduced methylviologen served as electron donor for assays with NAD^+^/NADP^+^ as substrate. Filled circles: NADH, open circles: NAD^+^, open boxes: NADPH, filled boxes: NADP^+^.

All three recombinant DsrL proteins appeared to be fully oxidized after purification under anoxic conditions. Addition of the strong oxidant ferricyanide [*E*^0^’ = + 0.418 in 50 mM phosphate buffer, pH 7.0 (O’Reilly, 1973)] did not lead to any alteration of the spectra. In all three cases complete reduction was achieved by addition of titanium(III) citrate [*E*^0^’ = −0.48 mV at pH 7.0 (Zehnder and Wuhrmann, 1976)] (Figures 5B–D). To obtain further insights into the properties of the Fe-S clusters, a redox titration was performed of the *Av*DsrL-1A protein in the presence of redox mediators, inside the anaerobic chamber starting with protein in as-isolated state and reducing with dithionite. The signal at *g* = 2.02 is masked by the presence of redox mediators and was not titratable by EPR. The EPR spectrum of fully reduced *Av*DsrL-1A at the end of the titration shows the same [4Fe-4S]^+^ rhombic signal observed in the sample without mediators. The intensity of the signal at *g* = 1.94 was followed during the redox titration (Figure 6C) in order to determine the redox potential of the centers. The signal only starts to appear below −270 mV, denoting a quite negative redox potential for the [4Fe-4S]^2+/+^ centers. The best fit to simulate the redox titration data with a Nernst equation was obtained by considering the sum of four centers with midpoint potentials (*E*_m_) of −330 mV and −390 mV in a 3:1 ratio, respectively, which agrees with the expected presence of four [4Fe-4S]^2+/+^ centers (Figure 6D).

*Av*DsrL-1A has previously been identified as NAD(P)H:acceptor oxidoreductase with strong preference for NADH over NADPH based on enzyme assays quantifying NAD(P)H oxidation with thiazolyl blue tetrazolium bromide (MTT) as electron acceptor (Löffler et al., 2020). Here, we add its kinetic characterization in the opposite direction, i.e., NAD(P)^+^ reduction with reduced methylviologen as electron donor. Just as in the NAD(P)H oxidizing direction, the affinity of the enzyme for the non-phosphorylated form of the nicotinamide cofactor is by far higher than for NADP^+^ (Figure 7A and Table 2). At the physiological pH of 7.0, *k*_cat_/*K*_M_ values amount to 1423 s^–1^ mM^–1^ and 57 s^–1^ mM^–1^ for the reactions with NAD^+^ and NADP^+^, respectively (Table 2). The much higher value for the NAD^+^-driven reaction clearly points at a strong preference of the *Av*DsrL-1A for NAD^+^ over NADP^+^ as a substrate under physiological conditions. *Ct*DsrL-1B from *C. tepidum* exhibited a temperature optimum of 40°C and worked well at pH 8.0. *C. tepidum* is known to grow optimally at 48–52°C and can also be cultured at ambient temperatures (Wahlund et al., 1991). DsrL-1B from *C. tepidum* did not show any activity with NADP^+^ or NADPH but was readily active with NAD^+^ and NADH (Figure 7B). Just the opposite was the case for the enzyme from *D. amilsii* (Figure 7C) which had a pH optimum at pH 6.5 and a temperature optimum at 45°C, consistent with optimal growth of the organism close to 50°C (Florentino et al., 2016). In sharp contrast to the two different DsrL-1-type enzymes, DsrL-2 from *D. amilsii* did not show any activity with NAD^+^ or NADH but turned out to be strictly dependent on NADPH or NADP^+^ as its substrates (Figure 7C and Table 2).

**TABLE 2:** Kinetic properties of *Allochromatium vinosum Av*DsrL-1A, *Chlorobaculum tepidum Ct*DsrL-1B and *Desulfurella amilsii Da*DsrL-2.

	***V*_max_ [U/mg]**	***K*_M_ [μM]**	***k*_cat_ [s**^–^**^1^]**	***k*_cat_/*K*_M_ [s**^–^**^1^mM**^–^**^1^]**
***Av*DsrL-1A**				
NADH	11.2 ± 0.6	86.7 ± 17.0	13.5	156
NADPH	4.0 ± 0.3	1667.0 ± 230.2	4.9	3
NAD^+^	144.9 ± 3.6	123.9 ± 10.3	176.3	1423
NADP^+^	120.1 ± 5.3	2563.0 ± 272.1	146.1	57
***Ct*DsrL-1B**				
NADH	436.0 ± 10.0	267.5 ± 20.5	460.2	1723
NAD^+^	366.5 ± 12.4	95.2 ± 9.9	386.8	4060
***Da*DsrL-2**				
NADPH	103.2 ± 2.5	124.5 ± 10.5	109.5	870
NADP^+^	88.8 ± 1.5	22.0 ± 1.9	94.3	4200

All three DsrL proteins studied here catalyze both the oxidation of a reduced nicotinamide cofactor and the reduction of the same oxidized cofactor at measurable rates. *Av*DsrL-1A displays significantly higher catalytic rates for NAD^+^ reduction than for NADH oxidation which indicates this enzyme’s bias to reductive catalysis (Figure 7A and Table 2) even if we acknowledge that two different redox dyes were used as electron acceptor/donor.

Close inspection of the structure for NfnB from *T. maritima* provides the basis for the different NAD(H) and NADP(H) specificity of the different DsrL enzymes studied here. In NfnB, the phosphate group of the nicotinamide cofactor is accommodated by Y^310^, R^311^ and R^312^ in a YRR motif (Demmer et al., 2015). The same series of amino acids is found at the respective position in *Da*DsrL-2 (Figure 3C) and in fact also in the other DsrLs of the same type with species of the *Candidatus* order Acidulodesulfobacterales as the only exceptions. Here, YRR is replaced by YNK (Figure 2). In the DsrL-1 enzymes, these residues are replaced by combinations of three amino acids taken from S/T-L/V/N/R/G/A/I-F/E/H/Q/Y/S/V/A which can be considered unsuitable for phosphate group binding. It thus appears that on the basis of kinetic characterization of model DsrL proteins and extrapolation on the basis of sequence motifs, DsrL-1 type enzymes are adapted to use NAD(H) as the substrate while enzymes of the DsrL-2 group are designed for the use of NADP(H).

## Discussion

We have shown previously that the iron-sulfur flavoprotein DsrL-1A from *A. vinosum* acts as physiological reaction partner for oxidative-type sulfite reductase, rDsrAB from the same organism (Löffler et al., 2020). *In vitro*, the rDsrABL-1A complex effectively catalyzes NADH-dependent sulfite reduction and thus NAD^+^ was identified as the probable *in vivo* electron acceptor for sulfur oxidation in organisms operating the rDsr pathway. The role of the low potential [4Fe-4S] clusters of DsrL in this reaction is currently enigmatic as it has been shown that the NADH-oxidizing activity of *Av*DsrL-1A and electron transfer from NADH to sulfite via *A. vinosum* rDsrABL-1A is not dependent on the presence of the iron-sulfur clusters (Löffler et al., 2020).

Here, we provide evidence that all organisms containing DsrL-1 have oxidative-type dissimilatory sulfite reductase. DsrL-1 enzymes are specifically adapted to use NAD^+^ and not NADP^+^ as electron acceptor. On the basis of our experimental results with the enzymes from *C. tepidum* and *A. vinsoum* we predict that all DsrL-1B enzymes are unable to replace NAD^+^ by NADP^+^ while more plasticity is present in DsrL-1A-type enzymes like the one from *A. vinosum*. The latter enzyme shows residual activity with NADP(H) (Figure 6A and Table 2).

When present, the DsrL enzymes from organisms containing bacterial reductive-type DsrAB fall within the DsrL-2 group and as experimentally shown for the model enzyme from *D. amilsii* they are all predicted to be active exclusively with NADP(H). In the context of a sulfate/sulfite reducer, DsrL-2 would thus be able to mediate electron transfer from NADPH but not from NADH to sulfite.

A number of organisms containing oxidative-type dissimilatory sulfite reductase feature an DsrL-2 enzyme reacting exclusively with NADP(H) (Figure 2). Two of the respective organisms are very well-established sulfur oxidizers (Imhoff and Thiel, 2010; Bryantseva et al., 2019). We conclude that in this case NADP^+^ is the electron acceptor for rDsrABL-2-catalyzed sulfite formation and that all organisms containing the rDsrABL-2 combination have the potential for sulfur oxidation. In principle, NADPH-driven sulfite reduction would also be possible in these organisms and cannot be excluded on the basis of sequence analyses alone without physiological characterization of the organisms. As already mentioned, organisms containing the rDsrABL-2 combination would be essentially unable to reduce NAD^+^ under sulfur-oxidizing conditions. This means that they would have to feed electrons into the more reduced pool of nicotinamide dinucleotide phosphate (Bennett et al., 2009) while this is not necessary in organisms confined to sulfur oxidation and containing a NAD^+^-reducing rDsrAB partner of the DsrL-1 type.

Together, our findings point to a functional evolution of DsrL resulting in adaption to the metabolic needs of the host organisms. Oxidative-type rDsrABL-1 and rDsrABL-2 combinations from sulfur oxidizers would act together in the transfer of electrons onto NAD^+^ and NADP^+^, respectively, while reductive-type DsrABL-2 complexes from sulfate/sulfite reducers require NADPH as electron donor and cannot operate with NADH. In bacterial cells, the NADP^+^/NADPH pool is generally maintained in a reduced state and NAD^+^/NADH in an oxidized state. The ratios can differ by several orders of magnitude depending on the organism and growth conditions. Reported NAD^+/^NADH values range from 3.74 to 31.3 whereas values range from 0.017 to 0.95 for NADP^+^/NADPH (Thauer et al., 1977; Bennett et al., 2009; Amador-Noguez et al., 2011; Spaans et al., 2015). The actual redox potential of both redox couples in living bacterial cells thus deviates significantly from the standard potential and is generally more negative than −320 mV for NADP^+^/NADPH and more positive than −320 mV for NAD^+^/NADH (Bennett et al., 2009; Buckel and Thauer, 2013; Spaans et al., 2015), which makes NAD^+^ a better electron acceptor than NADP^+^ while NADPH is a stronger reductant than NADH *in vivo.*

## Conclusion

In conclusion, our work adds to a framework allowing designation of metabolic types, in this case sulfur oxidizing and sulfur compound reducing capabilities. With confidence, organisms encoding enzymes of type DsrL-1 can be assigned as sulfur oxidizers. Organisms encoding DsrL-2 may either be sulfur oxidizers, sulfate/sulfite reducers or can switch between the two modes of energy conservation. On the basis of sequence data alone, it currently appears impossible to decide on their actual growth mode. In addition, the work of Thorup et al. (2017) has already shown that even the presence of a gene set interpreted as being typical for a sulfate reducer (no *dsrL*, no *dsrEFH*, reductive-type *dsrAB*) does not exclude oxidative sulfur metabolism. Together, these observations strongly emphasize the need for highly integrative approaches linking environmental sequence data with solid biochemical, physiological, biogeographical and geochemical data whenever possible. Given the highly mosaic nature of the various modules of Dsr-based oxidative and reductive sulfur metabolism, this is the one promising road when we strive for a valid picture of the natural sulfur cycle and the organisms driving it in the environment.

## Data Availability Statement

All datasets generated for this study are included in the article/Supplementary Material.

## Author Contributions

CD and ML designed the research. ML, KBW, and SSV performed the research. CD, ML, KBW, SSV, and IACP analyzed the data. CD and ML wrote the manuscript, which was approved by all authors.

## Conflict of Interest

The authors declare that the research was conducted in the absence of any commercial or financial relationships that could be construed as a potential conflict of interest.

## Supplementary Material

Supplementary Table S1 Occurrence of *dsrL* genes in bacterial genomes and metagenomes.

Supplementary Dataset S1 DsrA tree in Newick format.

Supplementary Dataset S2 DsrL tree in Newick format.
