# Erratum: The Iron-Sulfur Flavoprotein DsrL as NAD(P)H:Acceptor Oxidoreductase in Oxidative and Reductive Dissimilatory Sulfur Metabolism

**DOI:** 10.3389/fmicb.2020.644616

**Published:** 2021-01-13

**Authors:** Frontiers Production Office

**Affiliations:** Frontiers Media SA, Lausanne, Switzerland

**Keywords:** dissimilatory sulfate reduction, dissimilatory sulfur oxidation, DsrAB, DsrL, sulfur metabolism, sulfite reductase, NAD(P)H

Due to a production error, the incorrect Supplementary Material files were published. The Supplementary Material files have been corrected.

The publisher apologizes for this mistake. The original article has been updated.

