# Supplementary material for: The Iron-Sulfur Flavoprotein DsrL as NAD(P)H:Acceptor Oxidoreductase in Oxidative and Reductive Dissimilatory Sulfur Metabolism
Source: Front Microbiol. 2020 Oct 16;11:578209. doi: 10.3389/fmicb.2020.578209 (PMC7596348; doi:10.3389/fmicb.2020.578209)
Supplement: Supplementary Table S1 — Occurrence of dsrL genes in bacterial genomes and metagenomes. [file Data_Sheet_1.pdf]

Supplementary Information

The iron-sulfur flavoprotein DsrL as NAD(P)H: acceptor oxidoreductase in oxidative and reductive dissimilatory sulfur metabolism

Maria Löffler, Kai B. Wallerang, Christiane Dahl

Institut für Mikrobiologie & Biotechnologie, Rheinische Friedrich-Wilhelms-Universität Bonn, Bonn, Germany

Table S1. Occurrence of *dsrL* genes in bacterial genomes and metagenomes

For the following metagenomes Anantharaman et al. (Anantharaman et al., 2018) claimed the presence of DsrL, however, neither BlastP nor TBlastN searches in the respective Bioproject yielded results that matched our criteria: Chloroflexi bacterium RBG_13_60_13, Nitrospirae bacterium RIFOXYC2_FULL_44_7, Nitrospirae bacterium RBG_16_64_22, and Thermodesulfovibrio sp. RBG_19FT_COMBO_42_12, Nitrospinae bacteria RIFCSPLOWO2_02_39_17 and RIFCSPLOWO2_12_39_16, Candidatus Muproteobacteria bacteria RBG_16_60_9, RIFCSPHIGHO2_01_FULL_65_16, RBG_19FT_COMBO_61_10, RBG_16_65_34, and RBG_16_62_13 as well as Candidatus Rokubacteria bacteria GWA2_70_23, GWC2_70_24 and GWF2_70_14. TS, thiosulfate, TT, tetrathionate, HS^-, hydrogen sulfide, S^0, elemental sulfur, DMSP, dimethylsulfoniopropionate,

Taxonomic affiliation	Locus for DsrL	Locus tag	*dsrEFH*	Physiology	Reference
**Proteobacteria**					
**Alphaproteobacteria**					
**Rhizobiales**					
**Beijerinckiaceae**					
Rhodoblastus acidophilus DSM 137^T	WP_155444415	GJ654_02025	+	No photoautotrophic growth with HS^- or TS	(Imhoff, 2001b)
**Rhodobiaceae**					
Rhodobium orientis DSM 11290^T	RAI27273	CH339_10750	+	Photoautotrophic growth with TS	(Hiraishi et al., 1995; Imhoff, 2015a)
**Hyphomicrobiales**					
**Hyphomicrobiaceae**					
Rhodomicrobium vannielii ATCC 17100^T	ADP69871	Rvan_0592	+	Photoautotrophic growth with HS^-	(Brown et al., 2011; Imhoff, 2015a)
**Rhodospirillales**					
**Rhodospirillaceae**					
Magnetospirillum gryphiswaldense (DSM 6361^T) MSR-1	AVM74991	MSR1_25100	+	Autotrophic growth with HS^- or TS	(Geelhoed et al., 2010; Wang et al., 2014)

Magnetovibrio blakemorei DSM 18854^T (MV-1)	OEJ68387	BEN30_06410	+	Autotrophic growth with HS^- or TS	(Bazylinski et al., 2013b; Trubitsyn et al., 2016)
Phaeospirillum fulvum MGU-K5	SEH40517	SAMN04244559_02141	+	No photoautotrophic growth with HS^- or TS	(Imhoff, 2015b; Ivanovsky et al., 2017)
"Magnetospirillum magneticum" AMB-1	BAE52179	amb3375	+	Growth with HS^- or TS not tested	(Matsunaga et al., 2005)
**Rhodobacterales**					
**Rhodobacteraceae**					
Ruegeria marisrubri ZGT118^T (JCM 19519^T)	KUJ80512	AVO45_05545	+	Other species of Ruegeria degrade DMSP and oxidize HS^- and TS	(Moran et al., 2003; Todd et al., 2012; Zhang et al., 2017)
**Magnetococcales**					
**Magnetococcaceae**					
Magnetococcus marinus MC-1 (JCM 17883T)	ABK44659	Mmc1_2158	+	Autotrophic growth with HS^- or TS	(Bazylinski et al., 2013a)
**Betaproteobacteria**					
**Burkholdariales**					
**Burkholderiaceae**					
Burkholderiales bacterium JOSHI_001	EHR72136	BurJ1DRAFT_3327	+	Not tested	(Smith, 2014)
**Comamaonadaceae**					
Comamonadaceae bacterium SYSU G00088	WP_119289904	DRV96_RS08555	+	Unknown	Unpublished
**Nitrosomonadales**					
**Gallionellaceae**					
Sideroxydans lithotrophicus ES-1 (ATCC 700298^T)	ADE11911	Slit_1678	+	Growth on TS	(Emerson and Moyer, 1997; Emerson et al., 2013)
Sulfuricella denitrificans skB26 (DSM 22764^T)	BAN36510	SCD_n02710	+	Autotrophic growth with S^0 and TS	(Kojima and Fukui, 2010; Watanabe et al., 2014)
Sulfurirhabdus autotrophica DSM 100309^T	TCV86681	EDC63_10642	+	Autotrophic growth with S^0, TS and TT	(Watanabe et al., 2016; Watanabe et al., 2019)
**Sterolibacteriaceae**					
Sulfuritalea hydrogenivorans DSM 22779^T	BAO29235	SUTH_01436	+		(Watanabe et al., 2014)

**Thiobacillaceae**					
Thiobacillus denitrificans ATCC 25259	AAZ98430	Tbd_2477	+	Autotrophic sulfur oxidizer	(Beller et al., 2006)
Thiobacillus thioparus DSM 505^{T}	WP_026240625	B058_RS0104120	+	Autotrophic sulfur oxidizer	(Hutt et al., 2017)
**Gammaproteobacteria**					
**Chromatiales**					
**Chromatiaceae**					
Allochromatium vinosum DSM 180^{T}	AAG13082	Alvin_1259	+	Oxidizes HS^-, S^0, TS, sulfite	(Weissgerber et al., 2011)
Lamprocystis purpurea DSM 4197	WP_020504928	A39O_RS0112150	+	Oxidizes HS^-, S^0, TS	(Imhoff, 2001a)
Marichromatium purpuratum DSM 1591^{T}	AHF04296	MARPU_10875	+	Oxidizes HS^-, S^0, TS	(Imhoff et al., 1998)
Thermochromatium tepidum ATCC 43061^{T}	QGU33159	E6P07_09350	+	Oxidizes HS^-, S^0	(Madigan, 1986)
Thiocapsa marina DSM 5653^{T}	EGV17848	ThimaDRAFT_2907	+	Oxidizes HS^-, S^0, TS	(Caumette et al., 2004)
Thiocapsa rosea DSM 235^{T}	RKT45720	BDD21_3192	+	Oxidizes HS^-, S^0, TS	(Guyoneaud et al., 1998)
Thiocystis violascens DSM 198^{T}	AFL72611	Thivi_0552	+	Oxidizes HS^-, S^0, TS, sulfite	(Imhoff et al., 1998)
Thioflavicoccus mobilis DSM 8321^{T}	AGA89026	Thimo_0151	+	Oxidizes HS^-, S	(Imhoff and Pfennig, 2001)
Thiorhodococcus drewsii AZ1 DSM 15006^{T}	EGV31406	ThidrDRAFT_2028	+	Oxidizes HS^-, S^0, TS	(Zaar et al., 2003)
Thiorhodovibrio sp. 970	EIC21192	Thi970DRAFT_01381	+	Oxidizes HS^-, S^0, TS, sulfite	Unpublished
**Ectothiorhodospiraceae**					
Alkalilimnicola ehrlichii MLHE-1 (DSM 17681^{T})	ABI57007	Mlg_1661	+	Oxidizes HS^- and TS	(Hoeft et al., 2007)
Halorhodospira halophila DSM 244^{T}	ABM62723	Hhal_1959	+	Oxidizes HS^- and TS	(Challacombe et al., 2013)
Thioalkalivibrio nitratireducens DSM 14787^{T}	AGA33675	TVNIR_2014	+	Oxidizes HS^- and TS, much less active with S^0 and TT	(Berben et al., 2017)
Thioalkalivibrio halophilus HL17T (DSM 15791^{T})	OOC11187	B1A74_02005	+	Oxidizes HS^- and TS,	(Ahn et al., 2017)
Thioalkalivibrio sulfidiphilus HL-EbGr7 (NCCB 100376^{T})	ACL73273	Tgr7_2193	+	Oxidizes HS^-, TS, S^0 and TT	(Sorokin et al., 2012; Ahn et al., 2017)
Thioalkalivibrio thiocyanodenitrificans ARhD 1 (UNIQEM 226^{T})	WP_018232026	THITHI_RS0105235	+	Oxidizes HS^-, TS, S^0, TT and SCN^-	(Berben et al., 2015b)
Thioalkalivibrio paradoxus ARh 1 (DSM 13531T)	AHE98858	THITH_12020	+	Oxidizes HS^-, TS, S^0, CS_2 and SCN^-	(Sorokin et al., 2002; Berben et al., 2015a)
**Thiothrichales**					
**Thiotrichaceae**					
Thiothrix nivea DSM 5205^{T}	EIJ36276	Thini_3774	+	Oxidizes HS^-, TS, autotrophic growth questionable	(Lapidus et al., 2011)

Thiothrix caldifontis DSM 21228^{T}	SEA88964	SAMN05660964_02727	+	Autrophic growth on HS^{-} andTS,	(Chernousova et al., 2009)
Achromatium sp. WMS3	KOR32349	TI05_07780	+	Oxidize reduced sulfur compunds	(Mansor et al., 2015)
Thioploca ingrica	BAP54916	THII_0619	+	Oxidizes HS^{-}	(Kojima et al., 2015)
Candidatus Thiomargarita nelsonii	KHD06377	PN36_01220	+	Autotrophic growth on HS^{-}	(Flood et al., 2016)
**Thiolinaceae**					
Thiolinea disciformis DSM 14473^{T}	WP_020397038	A3IE_RS0118090	+	Oxidizes HS^{-} and TS during heterotrophic growth	(Boden and Scott, 2018)
**Thiofilaceae**					
Thiofilum flexile DSM 14609^{T}	WP_020560440	A3IK_RS0113080	+	Oxidizes HS^{-} and TS during heterotrophic growth	(Boden and Scott, 2018)
**Unclassified Gammaproteobacteria**					
Bathymodiolus sp. endosymbiont	WP_010645604	BSPSYM_RS00190	+	S-oxidizing endosymbiont	(Petersen et al., 2011)
Candidatus Ruthia magnifica *str. Cm*	ABL02578	Rmag_0862	+	S-oxidizing endosymbiont	(Newton et al., 2007)
Candidatus Vesicomyosocius okutanii HA	BAF61893	COSY_0787	+	S-oxidizing endosymbiont	(Kuwahara et al., 2007)
endosymbiont of *Riftia pachyptila* vent Ph05	EGV52269	Rifp1Sym_am00420	+	S-oxidizing endosymbiont	(Gardebrecht et al., 2012)
Solemya velesiana gill symbiont Sveles-Q1	OOZ37468	BOW51_02115	+	S-oxidizing endosymbiont	(Russell et al., 2017)
Sedimenticola selenatireducens BM301	PLX61201	C0630_12470	+	Autotrophic growth with TS, HS^{-} and TT	(Barnum et al., 2018)
Thiolapillus brandeum Hiromi 1 (DSM 23672^{T})	BAO45363	TBH_C2454	+	Autotrophic growth with TS, HS^{-} and TT	(Nunoura et al., 2014)
Gammaproteobacteria bacterium RIFOXYD12_FULL_61_37	OGT90902	A2514_07170	+	unknown	(Anantharaman et al., 2016)
**Deltaproteobacteria**					
**Desulfurellales**					
**Desulfurellaceae**					
Desulfurella amilsii DSM 29984^{T}	OSS41879	DESAMIL20_1432	-	Reduces S^{0} and TS, S^{0} disproportionation	(Florentino et al., 2017)
Desulfurella multipotens DSM 8415^{T}	SDB96229	SAMN05660835_00065	-	Reduces S^{0}	(Miroshnichenko et al., 1994)
**Desulfbacterales**					
**Desulfbacteraceae**					
Desulfobacteraceae bacterium isolate E44_bin91	TES94602	E3J94_00320	-	Unknown	Dong and Hubert, unpublished

Desulfobacter sp .isolate ARS36	MAF33455	CL941_05810	-	Unkown	(Tully et al., 2018)
**Desulfobulbaceae**					
Desulforhopalus sp. IMCC35007	WP_136875449	FCL48_21255	-	Unkown	Hwang, unpublished
Desulfopila sp. IMCC35006	WP_167496548	FCL47_RS17550		Unkown	Hwang, unpublished
Desulfopila sp. IMCC35008	WP_163338802	GA520_RS15900	-	Unkown	Hwang, unpublished
Desulfobulbaceae bacterium S3730MH12	OEU56217	BA862_03835	-	Unkown	Skennerton and Orphan, unpublished
Desulfobulbaceae bacterium S5133MH15 k99_1319126	MAXK01000062	BA866_07805	-	Unknown	Skennerton and Orphan, unpublished
**Unclassified Desulfobacterales**					
Desulfobacterales bacterium HyVt-363	HEA67260	ENI07_10635	-	Unknown	(Zhou et al., 2020)
**Myxococcales**					
Myxococcales bacterium SURF_8	RJO72419	C4523_03280	+	Unknown	(Momper et al., 2017)
Myxococcales bacterium SURF_8	RJO72418	C4523_03275		Unknown	(Momper et al., 2017)
Anaeromyxobacter sp. RBG_16_69_14	OFX25127	A2V77_06045	-	Unknown	(Anantharaman et al., 2016; Anantharaman et al., 2018)
**Unclassified Deltaproteobacteria**					
Candidatus Acididesulfobacter diazotrophicus AP1	RZD18491	EVG15_05520	+	Likely sulfate reducers, possibly also HS^- oxidation	(Tan et al., 2019)
Candidatus Acidulodesulfobacterium acidiphilum AP4	RZV38614	EVJ48_06505	+	Likely sulfate reducers, possibly also HS^- oxidation,	(Tan et al., 2019)
Candidatus Acidulodesulfobacterium ferriphilum AP3	RZD14989	EVJ47_01545	+	Likely sulfate reducers, possibly also HS^- oxidation	(Tan et al., 2019)
Deltaproteobacteria bacterium isolate SpSt-825	HHS65189	ENW17_08270	-		(Zhou et al., 2020)
Deltaproteobacteria bacteria isolate NP955	MP45434	CL925_13695	+	Unknown	(Tully et al., 2018)
Deltaproteobacteria bacterium isolate UBA12577	HCV44982	DF909_02655	+	Unknown	(Parks et al., 2018)
Deltaproteobacteria bacterium isolate UBA9332	HBR59122	DEA86_01430	+	Unknown	(Parks et al., 2018)
Deltaproteobacteria bacterium isolate UBA9617	HCP34911	DIT94_11020	+	Unknown	(Parks et al., 2018)
Deltaproteobacteria bacterium B64_G9	RLB58993	DRI34_03225	-	Unknown	(Dombrowski et al., 2018)
Deltaproteobacteria bacterium ADurb.Bin207	OQB11090	BWY17_04067	-	Unknown	Nobu, et al, unpublished
Deltaproteobacteria bacterium isolate UBA8081	HAF89896	DCG23_08995	+	Unknown st	(Parks et al., 2018)
Deltaproteobacteria bacterium isolate CG_2015-19_70_48	NCO58380	GW870_09430	+	Unknown	(Probst et al., 2020)

Deltaproteobacteria bacterium RIFOXYA12_FULL_61_11	A2284_04105	OGR01626	-	Unknown	(Anantharaman et al., 2016; Anantharaman et al., 2018)
**Lambdaproteobacteria**					
Candidatus_Lambdaproteobacteria_bacterium_RIFOXYD2_FULL_50_16	OGG97050	A2527_03055	+	Proposed: sulfite reduction, sulfur oxidation or both	(Anantharaman et al., 2016; Anantharaman et al., 2018)
Candidatus_Lambdaproteobacteria_bacterium_RIFOXYC1_FULL_56_13	OGH01168	A2426_01665	+	Proposed: sulfite reduction, sulfur oxidation or both	(Anantharaman et al., 2016; Anantharaman et al., 2018)
***Candidatus* Muproteobacteria**					
Candidatus Muproteobacteria bacterium RBG_16_65_31	OGI43538	A2V92_03640	+	Proposed sulfur oxidation	(Anantharaman et al., 2016; Anantharaman et al., 2018)
Candidatus Muproteobacteria bacterium RIFCSPHIGHO2_01_FULL_61_200	OGI57755	A2809_07060	+	Proposed sulfur oxidation	(Anantharaman et al., 2016; Anantharaman et al., 2018)
Candidatus Muproteobacteria bacterium RIFCSPLOWO2_01_FULL_60_18	OGI50061	A3A87_05115	+	Proposed sulfur oxidation	(Anantharaman et al., 2016; Anantharaman et al., 2018)
Candidatus Muproteobacteria bacterium RIFCSPHIGHO2_02_FULL_65_16	OGI51488	A3B81_01765	+	Proposed sulfur oxidation	(Anantharaman et al., 2016; Anantharaman et al., 2018)
Candidatus Muproteobacteria bacterium RIFCSPHIGHO2_02_FULL_60_13	OGI56109	A3D32_00265	+	Proposed sulfur oxidation	(Anantharaman et al., 2016; Anantharaman et al., 2018)
Candidatus Muproteobacteria bacterium RIFCSPHIGHO2_12_FULL_60_33	OGI55112	A3E57_06600	+	Proposed sulfur oxidation	(Anantharaman et al., 2016; Anantharaman et al., 2018)
**Hydrogenophilalia**					
**Hydrogenophilales**					
**Hydrogenophilaceae**					
Hydrogenophilaceae bacterium L.E.AP.33	NWG39505	HXY27_06025	+	Unknown, some described family members oxidize sulfur	Levy-Booth, D.J., unpublished, (Orlygsson and

					Kristjansson, 2014; Boden et al., 2017)
					Levy-Booth, D.J., unpublished,
Hydrogenophilaceae bacterium L.E.AP.25	NWG86727	HXY26_04320	+	Unknown, some described family members oxidize sulfur	(Orlygsson and Kristjansson, 2014; Boden et al., 2017)
Hydrogenophilaceae bacterium CG1_02_62_390	OIO76938	AUJ86_10720	+	Unknown, some described family members oxidize sulfur	(Orlygsson and Kristjansson, 2014; Boden et al., 2017; Probst et al., 2017)
**Unclassified Hydrogenophilales**					
Hydrogenophilales bacterium 17-64-11	OZA28583	B7X2_04930	+	Unknown, some described members of the order oxidize sulfur	Kantor, R. S. et al unpublished, (Boden et al., 2017)
Hydrogenophilales bacterium 12-64-13	OYW66411	B7X91_01055	+	Unknown, some described members of the order oxidize sulfur	Kantor, R. S. et al unpublished, (Boden et al., 2017)
Hydrogenophilales bacterium 16-64-40	OYZ29725	B7Y27_03045	+	Unknown, some described members of the order oxidize sulfur	Kantor, R. S. et al unpublished, (Boden et al., 2017)
Hydrogenophilales bacterium 20-61-23	OYY93365	B7Y41_11335	+	Unknown, some described members of the order oxidize sulfur	Kantor, R. S. et al unpublished, (Boden et al., 2017)
Hydrogenophilales bacterium 12-61-10	OYW37845	B7Z35_08735	+	Unknown, some described members of the order oxidize sulfur	Kantor, R. S. et al unpublished, (Boden et al., 2017)
Hydrogenophilales bacterium 16-61-112	OYZ57360	B7Y21_07885	+	Unknown, some described members of the order oxidize sulfur	Kantor, R. S. et al unpublished, (Boden et al., 2017)
Hydrogenophilales bacterium RIFOXYD1_FULL_62_11	OGU21535	A2580_15765	+	Unknown, some described members of the order oxidize sulfur	(Anantharaman et al., 2016; Boden et al., 2017)
Hydrogenophilales bacterium CG_4_10_14_3_um_filter_63_21	PIX98389	COZ24_00400	+	Unknown, some described members of the order oxidize sulfur	(Boden et al., 2017; Probst et al., 2018)
Hydrogenophilales bacterium CG18_big_fil_WC_8_21_14_2_50_58_12	PIQ09875	COW70_14675	+	Unknown, some described members of the order oxidize sulfur	(Boden et al., 2017; Probst et al., 2018)

**Acidobacteria**					
**Acidobacteriia**					
**Unclassified Acidobacteriia**					
Acidobacteria bacterium 21-70-11	OYV97919	B7Z68_02320	-	Proposed: sulfate/sulfite reduction or sulfur oxidation or both	(Anantharaman et al., 2018)
Acidobacteria bacterium 37-71-11	OYW06934	B7Z61_00725	-	Proposed: sulfate/sulfite reduction or sulfur oxidation or both	(Anantharaman et al., 2018)
Acidobacteriia bacterium SbA2	SPE28132	SBA2_380041	-	Proposed: sulfate reduction, possibly also HS^- oxidation and S^0 disproportionation	(Hausmann et al., 2018)
**Acidobacteriales**					
**Acidobacteriaceae**					
Candidatus Sulfotelmatobacter kueseliae MAG SbA1	SPF32259	SBA1_1040025	-	Proposed sulfite and organosulfonate reduction, possibly also HS^- oxidation and S^0 disproportionation	(Hausmann et al., 2018)
**Bryobacterales**					
**Solibacteraceae**					
Candidatus Sulfopaludibacter sp. SbA3	SPE43172	SBA3_840004	-	Proposed sulfate reduction, possibly also HS^- oxidation and S^0 disproportionation	(Hausmann et al., 2018)
**Terrabacteria group**					
**Actinobacteria**					
**Unclassified Actinobacteria**					
Actinobacteria bacterium CG2_30_50_142	OIO86557	AUK32_05245	+	Unknown	(Anantharaman et al., 2016; Anantharaman et al., 2018)
Actinobacteria bacterium CG_4_10_14_3_um_filter_50_156	PIY39369	COZ03_06260	+	Unknown	(Probst et al., 2018)
Actinobacteria bacterium CG08_land_8_20_14_0_20_50_39	PIU27534	COT10_02975	+	Unknown	(Probst et al., 2018)

**Rubrobacteria**					
**Gaeillales**					
Gaiellales bacterium isolate SURF_19	RJQ47444	C4534_01030	+	Unknown	(Momper et al., 2017)
Gaiellales bacterium isolate SURF_19	RJQ47451	C4534_01065	+	Unknown	(Momper et al., 2017)
**Chloroflexi**					
**Thermomicrobia**					
**Thermomicrobiales**					
Thermomicrobiales bacterium Bin_2_1 (wastewater metagenome)	TXG81329	E6R14_07885	+.	Unknown	(Stamps et al., 2018)
**Armatimonadetes**					
Armatimonadetes bacterium CG_4_9_14_3_um_filter_66_14	PJB60213	CO096_35085	-	Unknown	(Probst et al., 2018)
Armatimonadetes bacterium CG2_30_66_41	OIP09157	AUJ96_05550	-	Proposed: sulfate/sulfite reduction	(Anantharaman et al., 2016; Anantharaman et al., 2018)
Armatimonadetes bacterium CG_4_8_14_3_um_filter_66_20	PIX40392	COZ57_26040	-	Unknown	(Probst et al., 2018)
**FCB group**					
**Candidate division Zixibacteria**					
Candidate division Zixibacteria bacterium SURF_9	RJP73754	C4524_13795	-	Proposed sulfate/sulfite reduction	(Momper et al., 2017)
Candidate division Zixibacteria bacterium HGW-Zixibacteria-1	PKK84342	CVT49_04215	-	Proposed sulfur oxidation	(Hernsdorf et al., 2017)
Candidate division Zixibacteria bacterium isolate SpSt-1246	HER00049	ENO22_11980	-	Unknown	(Zhou et al., 2020)
Candidate division Zixibacteria bacterium isolate HyVt-29	HDL03316	ENH25_04205	-	Unknown	(Zhou et al., 2020)
Candidate division Zixibacteria bacterium isolate KS3-K099	NIP41367	GWO28_01305	-	Unknown	Chandrashekar et al, unpublished
**Gemmatimonadetes**					
**Gemmatimonadales**					
**Unclassified Gemmatimonadales**					
Gemmatimonadales bacterium das_tool.cocacola.36	TFH67021	E4G90_00505	-	Unknown	(Rasigraf et al., 2019)

**Bacteroidetes/Chlorobi group**					
**Chlorobi**					
**Chlorobia**					
**Chlorobiales**					
**Chlorobiaceae**					
Chlorobaculum limnaeum DSM 1677T	AOS84661	BIU88_11275	+	HS^- oxidized	(Imhoff, 2014; Tank et al., 2017)
Chlorobaculum limnaeum DSM 1677T	AOS83892	BIU88_06830	+	HS^- oxidized	(Imhoff, 2014; Tank et al., 2017)
Chlorobaculum parvum DSM 263T	ACF10458	Cpar_0030	-	TS oxidized, HS^- oxidized to S^0	(Kelly, 2008; Gregersen et al., 2011)
Chlorobaculum tepidum ATCC 49652T	AAM73463	CT2247	+	HS^-, TS oxidized	(Imhoff, 2003)
Chlorobaculum tepidum ATCC 49652T	AAM72090	CT0854	+	HS^-, TS oxidized	(Imhoff, 2003)
Chlorobium chlorochromatii CaD3	ABB29202	Cag_1954	+	HS^- oxidized	(Vogl et al., 2006; Davenport et al., 2010; Imhoff, 2014)
Chlorobium limicola DSM 245T	ACD89779	Clim_0696	+	HS^-, TS (±) oxidized	(Imhoff, 2003; 2014)
Chlorobium limicola Frassassi	KUL33300	ASB62_00360	+	HS^-, TS oxidized	(Mansor and Macalady, 2016)
Chlorobium luteolum DSM 273T	ABB22928	Plut_0038	+	HS^- oxidized	(Imhoff, 2003)
Chlorobium phaeobacteroides DSM 266T	ABL64200	Cpha266_0130	+	HS^- oxidized	(Imhoff and Thiel, 2010; Imhoff, 2014)
Chlorobium phaeobacteroides BS1	ACE04772	Cphamn1_1855	+	HS^- oxidized	(Imhoff and Thiel, 2010)
Chlorobium phaeovibrioides DSM 265	ABP36069	Cvib_0041	+	HS^- oxidized	(Imhoff and Thiel, 2010)
Pelodictyon phaeoclathratiforme BU-1 (DSM 5477T)	ACF44514	Ppha_2319	+	HS^-, S^0, TS oxidized	(Overmann and Pfennig, 1989)
Prosthecochloris aestuarii DSM 271T	ACF45097	Paes_0031	+	HS^- oxidized	(Imhoff and Thiel, 2010)
Prosthecochloris sp. GSB1	ASQ89562	CHL67_00165	+	HS^- oxidized	(Imhoff and Thiel, 2010; Bryant et al., 2012)
Prosthecochloris marina V1 (KCTC 15824T)	PWW81152	CR164_11435	+	HS^-, TS oxidized	(Bryantseva et al., 2019)
**Unclassified Bacteroidetes/Chlorobi group**					
Bacteroidetes/Chlorobi group bacterium ChocPot_Midway_bin-8	ROL58764	D9V86_11585	-	Proposed sulfate reduction	(Thiel et al., 2019)
Bacteroidetes/Chlorobi group bacterium MS-B_bin-24	ROL57978	D9V87_08935	-	Proposed sulfate reduction	(Thiel et al., 2019)
Bacteroidetes/Chlorobi group bacterium Naka2016	ROL57725	D9V84_03110	-	Proposed sulfate reduction	(Thiel et al., 2019)

**Nitrospinae/Tectomicrobia group**					
**Nitrospinae**					
**Unclassified Nitrospinae**					
Nitrospinae bacterium isolate UBA9963	HBA26581	DCY98_04200	+	Unknown	(Parks et al., 2018)
**Nitrospirae**					
**Unclassified Nitrospirae**					
Nitrospirae bacterium CG2_30_53_67	OIP62037	AUK29_09310	+	Proposed sulfur oxidation	(Probst et al., 2017; Anantharaman et al., 2018)
Nitrospirae bacterium CG2_30_53_67	OIP65749	AUK29_02145	+	Proposed sulfur oxidation	(Probst et al., 2017; Anantharaman et al., 2018)
Nitrospirae bacterium CG2_30_70_394	OIP63244	AUK30_08860	+	Unknown	(Probst et al., 2017)
Nitrospirae bacterium RBG_19FT_COMBO_42_15	OGW50294	A2Z50_01630	-	Proposed sulfate reduction	(Anantharaman et al., 2016; Anantharaman et al., 2018)
Nitrospirae bacterium CG_4_10_14_3_um_filter_50_41	PIW85965	COZ95_01815	+	Unknown	(Probst et al., 2018)
Nitrospirae bacterium CG08_land_8_20_14_0_20_52_24	PIS37301	COT35_06715	+	Unknown	(Probst et al., 2018)
Nitrospirae bacterium CG17_big_fil_post_rev_8_21_14_2_50_50_9	PIV84583	COW52_06785	+	Unknown	(Probst et al., 2018)
**PVC group**					
**Planctomycetes**					
**unclassified Planctomycetes**					
Planctomycetes bacterium RBG_13_63_9	OHB72513	A2V70_00910	-	Sulfate/sulfite reduction	(Anantharaman et al., 2018; Probst et al., 2018)
**Ignavibacteriae**					
**Unclassified Ignavibacteriae**					
Ignavibacteriae bacterium B3_G1 (DsrA gene is split on different contigs)	RKY97480	DRQ13_04645		Unknown	(Dombrowski et al., 2018)

Ignavibacteria bacterium GWB2_35_12	OGU41173	A2X61_13720	-	Proposed sulfate/ sulfite reduction	(Anantharaman et al., 2016; Anantharaman et al., 2018)
Ignavibacteria bacterium GWA2_35_8	OGU14715	A2X63_09505	-	Proposed sulfate/sulfite reduction	(Anantharaman et al., 2016; Anantharaman et al., 2018)
Ignavibacteria bacterium RIFOXYA2_FULL_35_10	OGU89118	A2220_15450	-	Proposed sulfate/ sulfite reduction	(Anantharaman et al., 2016; Anantharaman et al., 2018)
Ignavibacteria bacterium RIFOXYC2_FULL_35_21	OGV23095	A2475_17050	-	Proposed sulfate/ sulfite reduction	(Anantharaman et al., 2016; Anantharaman et al., 2018)
**Verrucomicrobia**					
**Methylacidiphilae**					
**Methylacidiphilales**					
Methylacidiphilales bacterium isolate SM1_5_30	NJL06915	HC900_00610		Unknown	(Waterworth et al., 2020)
**Verrucomicrobiae**					
**Verrucomicrobiales**					
Verrucomicrobiales bacterium isolate NAT181	MAW12575	CMO54_12140	+	Unknown	(Tully et al., 2018)
**Bacteria candidate phyla**					
**Candidatus Schekmanbacteria**					
Candidatus Schekmanbacteria bacterium RBG_13_48_7	OGL46692	A2161_21820	-	Proposed sulfate/sulfite reduction	(Anantharaman et al., 2016; Anantharaman et al., 2018)
**Candidatus Desantisbacteria**					
Candidatus Desantisbacteria bacterium CG2_30_40_21	OIP40282	AUJ95_04735	-	Proposed sulfite/sulfate reduction	(Probst et al., 2017; Anantharaman et al., 2018)
Candidatus Desantisbacteria bacterium CG_4_8_14_3_um_filter_40_12	PIX17661	COZ71_02200	-	Unknown	(Probst et al., 2018)

**Candidatus Omnitrophica**					
Candidatus Omnitrophica bacterium SURF12	RJP18718	C4527_28415	-	Unknown	(Momper et al., 2017)
**Unclassified Bacteria**					
Uncultured sulfate-reducing bacterium	CAJ31166	39f70008	-	Proposed sulfate reduction	(Mussmann et al., 2005)

References

Ahn, A. C., Meier-Kolthoff, J. P., Overmars, L., Richter, M., Woyke, T., Sorokin, D. Y., et al. (2017). Genomic diversity within the haloalkaliphilic genus *Thioalkalivibrio*. *PLoS One* 12**,** e0173517. doi: 10.1371/journal.pone.0173517.

Anantharaman, K., Brown, C. T., Hug, L. A., Sharon, I., Castelle, C. J., Probst, A. J., et al. (2016). Thousands of microbial genomes shed light on interconnected biogeochemical processes in an aquifer system. *Nat. Commun.* 7**,** 13219. doi: 10.1038/ncomms13219.

Anantharaman, K., Hausmann, B., Jungbluth, S. P., Kantor, R. S., Lavy, A., Warren, L. A., et al. (2018). Expanded diversity of microbial groups that shape the dissimilatory sulfur cycle. *ISME J.* 12**,** 1715-1728. doi: 10.1038/s41396-018-0078-0.

Barnum, T. P., Figueroa, I. A., Carlstrom, C. I., Lucas, L. N., Engelbrektson, A. L., and Coates, J. D. (2018). Genome-resolved metagenomics identifies genetic mobility, metabolic interactions, and unexpected diversity in perchlorate-reducing communities. *ISME J.* 12**,** 1568-1581. doi: 10.1038/s41396-018-0081-5.

Bazylinski, D. A., Williams, T. J., Lefevre, C. T., Berg, R. J., Zhang, C. L., Bowser, S. S., et al. (2013a). *Magnetococcus marinus* gen. nov., sp. nov., a marine, magnetotactic bacterium that represents a novel lineage (*Magnetococcaceae* fam. nov., *Magnetococcales* ord. nov.) at the base of the *Alphaproteobacteria*. *Int. J. Syst. Evol. Microbiol.* 63**,** 801-808. doi: 10.1099/ijs.0.038927-0.

Bazylinski, D. A., Williams, T. J., Lefevre, C. T., Trubitsyn, D., Fang, J., Beveridge, T. J., et al. (2013b). *Magnetovibrio blakemorei* gen. nov., sp. nov., a magnetotactic bacterium (Alphaproteobacteria: Rhodospirillaceae) isolated from a salt marsh. *Int. J. Syst. Evol. Microbiol.* 63**,** 1824-1833. doi: 10.1099/ijs.0.044453-0.

Beller, H. R., Chai, P. S. G., Letain, T. E., Chakicherla, A., Larimer, F. W., Richardson, P. M., et al. (2006). The genome sequence of the obligately chemolithoautotrophic, facultatively anaerobic bacterium *Thiobacillus denitrificans*. *J. Bacteriol.* 188**,** 1473-1488. doi: 10.1128/JB.188.4.1473-1488.2006.

Berben, T., Overmars, L., Sorokin, D. Y., and Muyzer, G. (2017). Comparative genome analysis of three thiocyanate oxidizing *Thioalkalivibrio* species isolated from soda lakes. *Front. Microbiol.* 8**,** 254. doi: 10.3389/fmicb.2017.00254.

Berben, T., Sorokin, D. Y., Ivanova, N., Pati, A., Kyrpides, N., Goodwin, L. A., et al. (2015a). Complete genome sequence of *Thioalkalivibrio paradoxus* type strain ARh 1^T, an obligately chemolithoautotrophic haloalkaliphilic sulfur-oxidizing bacterium isolated from a Kenyan soda lake. *Stand. Genomic Sci.* 10**,** 105. doi: 10.1186/s40793-015-0097-7.

Berben, T., Sorokin, D. Y., Ivanova, N., Pati, A., Kyrpides, N., Goodwin, L. A., et al. (2015b). Partial genome sequence of *Thioalkalivibrio thiocyanodenitrificans* ARhD 1^T, a chemolithoautotrophic haloalkaliphilic sulfur-oxidizing bacterium capable of complete denitrification. *Stand. Genomic Sci.* 10**,** 84. doi: 10.1186/s40793-015-0080-3.

Boden, R., Hutt, L. P., and Rae, A. W. (2017). Reclassification of *Thiobacillus aquaesulis* (Wood & Kelly, 1995) as *Annwoodia aquaesulis* gen. nov., comb. nov., transfer of *Thiobacillus* (Beijerinck, 1904) from the *Hydrogenophilales* to the *Nitrosomonadales*, proposal of *Hydrogenophilalia* class. nov. within the 'Proteobacteria', and four new families within the orders *Nitrosomonadales* and *Rhodocyclales*. *Int. J. Syst. Evol. Microbiol.* 67**,** 1191-1205. doi: 10.1099/ijsem.0.001927.

Boden, R., and Scott, K. M. (2018). Evaluation of the genus *Thiothrix* Winogradsky 1888 (Approved Lists 1980) emend. Aruga et al. 2002: reclassification of *Thiothrix disciformis* to *Thiolinea disciformis* gen. nov., comb. nov., and of *Thiothrix flexilis* to *Thiofilum flexile* gen. nov., comb nov., with emended description of *Thiothrix*. *Int. J. Syst. Evol. Microbiol.* 68**,** 2226-2239. doi: 10.1099/ijsem.0.002816.

Brown, P. J., Kysela, D. T., Buechlein, A., Hemmerich, C., and Brun, Y. V. (2011). Genome sequences of eight morphologically diverse Alphaproteobacteria. *J. Bacteriol.* 193**,** 4567-4568. doi: 10.1128/JB.05453-11.

Bryant, D. A., Liu, Z., Li, T., Zhao, F., and Garcia Costas, A. M. (2012). "Comparative and functional genomics of anoxygenic green bacteria from the taxa *Chlorobi*, *Chloroflexi*, and *Acidobacteria*," in *Functional genomics and evolution of photosynthetic systems,* eds. R. Burnap & W. Vermass. (Dordrecht: Springer), 47-102.

Bryantseva, I. A., Tarasov, A. L., Kostrikina, N. A., Gaisin, V. A., Grouzdev, D. S., and Gorlenko, V. M. (2019). *Prosthecochloris marina* sp. nov., a new green sulfur bacterium from the coastal zone of the South China Sea. *Arch. Microbiol.* 201**,** 1399-1404. doi: 10.1007/s00203-019-01707-y.

Caumette, P., Guyoneaud, R., Imhoff, J. F., Süling, J., and Gorlenko, V. (2004). *Thiocapsa marina* sp. nov., a novel, okenone-containing, purple sulfur bacterium isolated from brackish coastal and marine environments. *Int. J. Syst. Evol. Microbiol.* 54**,** 1031-1036. doi: 10.1099/ijs.0.02964-0.

Challacombe, J. F., Majid, S., Deole, R., Brettin, T. S., Bruce, D., Delano, S. F., et al. (2013). Complete genome sequence of *Halorhodospira halophila* SL1. *Stand. Genomic Sci.* 8**,** 206-214. doi: 10.4056/sigs.3677284.

Chernousova, E., Gridneva, E., Grabovich, M., Dubinina, G., Akimov, V., Rossetti, S., et al. (2009). T*hiothrix caldifontis* sp. nov. and *Thiothrix lacustris* sp. nov., gammaproteobacteria isolated from sulfide springs. *Int. J. Syst. Evol. Microbiol.* 59**,** 3128-3135. doi: 10.1099/ijs.0.009456-0.

Davenport, C., Ussery, D. W., and Tummler, B. (2010). Comparative genomics of green sulfur bacteria. *Photosynth. Res.* 104**,** 137-152. doi: 10.1007/s11120-009-9515-2.

Dombrowski, N., Teske, A. P., and Baker, B. J. (2018). Expansive microbial metabolic versatility and biodiversity in dynamic Guaymas Basin hydrothermal sediments. *Nat. Commun.* 9**,** 4999. doi: 10.1038/s41467-018-07418-0.

Emerson, D., Field, E. K., Chertkov, O., Davenport, K. W., Goodwin, L., Munk, C., et al. (2013). Comparative genomics of freshwater Fe-oxidizing bacteria: implications for physiology, ecology, and systematics. *Front. Microbiol.* 4**,** 254. doi: 10.3389/fmicb.2013.00254.

Emerson, D., and Moyer, C. (1997). Isolation and characterization of novel iron-oxidizing bacteria that grow at circumneutral pH. *Appl. Environ. Microbiol.* 63**,** 4784-4792. doi: 10.1128/Aem.63.12.4784-4792.1997.

Flood, B. E., Fliss, P., Jones, D. S., Dick, G. J., Jain, S., Kaster, A. K., et al. (2016). Single-cell (meta-)genomics of a dimorphic *Candidatus* Thiomargarita nelsonii reveals genomic plasticity. *Front. Microbiol.* 3**,** 602. doi: 10.3389/fmicb.2016.00603.

Florentino, A. P., Stams, A. J., and Sanchez-Andrea, I. (2017). Genome sequence of *Desulfurella amilsii* Strain TR1 and comparative genomics of Desulfurellaceae family. *Front. Microbiol.* 8**,** 222. doi: 10.3389/fmicb.2017.00222.

Gardebrecht, A., Markert, S., Sievert, S. M., Felbeck, H., Thürmer, A., Albrecht, D., et al. (2012). Physiological homogeneity among the endosymbionts of *Riftia pachyptila* and *Tevnia jerichonana* revealed by proteogenomics. *ISME J.* 6**,** 766-776. doi: 10.1038/ismej.2011.137.

Geelhoed, J. S., Kleerebezem, R., Sorokin, D. Y., Stams, A. J., and van Loosdrecht, M. C. (2010). Reduced inorganic sulfur oxidation supports autotrophic and mixotrophic growth of *Magnetospirillum* strain J10 and *Magnetospirillum gryphiswaldense*. *Environ. Microbiol.* 12**,** 1031-1040. doi: 10.1111/j.1462-2920.2009.02148.x.

Gregersen, L. H., Bryant, D. A., and Frigaard, N. U. (2011). Mechanisms and evolution of oxidative sulfur metabolism in green sulfur bacteria. *Front. Microbiol.* 2**,** 116. doi: 10.3389/fmicb.2011.00116.

Guyoneaud, R., Süling, J., Petri, R., Matheron, R., Caumette, P., Pfennig, N., et al. (1998). Taxonomic rearrangements of the genera *Thiocapsa* and *Amoebobacter* on the basis of 16S rDNA sequence analyses, and description of *Thiolamprovum* gen. nov. *Int. J. Syst. Bacteriol.* 48**,** 957-964. doi: 10.1099/00207713-48-3-957.

Hausmann, B., Pelikan, C., Herbold, C. W., Kostlbacher, S., Albertsen, M., Eichorst, S. A., et al. (2018). Peatland Acidobacteria with a dissimilatory sulfur metabolism. *ISME J.* 12**,** 1729-1742. doi: 10.1038/s41396-018-0077-1.

Hernsdorf, A. W., Amano, Y., Miyakawa, K., Ise, K., Suzuki, Y., Anantharaman, K., et al. (2017). Potential for microbial H_2 and metal transformations associated with novel bacteria and archaea in deep terrestrial subsurface sediments. *ISME J.* 11**,** 1915-1929. doi: 10.1038/ismej.2017.39.

Hiraishi, A., Urata, K., and Satoh, T. (1995). A new genus of marine budding phototrophic bacteria, *Rhodobium* gen. nov., which includes *Rhodobium orientis* sp. nov. and *Rhodobium marinum* comb. nov. *Int. J. Syst. Bacteriol.* 45**,** 226-234. doi: 10.1099/00207713-45-2-226.

Hoeft, S. E., Blum, J. S., Stolz, J. F., Tabita, F. R., Witte, B., King, G. M., et al. (2007). *Alkalilimnicola ehrlichii* sp. nov., a novel, arsenite-oxidizing haloalkaliphilic gammaproteobacterium capable

of chemoautotrophic or heterotrophic growth with nitrate or oxygen as the electron acceptor. *Int. J. Syst. Evol. Microbiol.* 57**,** 504-512. doi: 10.1099/ijs.0.64576-0.

Hutt, L. P., Huntemann, M., Clum, A., Pillay, M., Palaniappan, K., Varghese, N., et al. (2017). Permanent draft genome of *Thiobacillus thioparus* DSM 505^T, an obligately chemolithoautotrophic member of the Betaproteobacteria. *Stand. Genomic Sci.* 12**,** 10. doi: 10.1186/s40793-017-0229-3.

Imhoff, J. F. (2001a). Transfer of *Pfennigia purpurea* Tindall 1999 (A*moebobacter purpureus* Eichler and Pfennig 1988) to the genus *Lamprocystis* as *Lamprocystis purpurea* comb. nov. *Int. J. Syst. Evol. Microbiol.* 51**,** 1699-1701. doi: Doi 10.1099/00207713-51-5-1699.

Imhoff, J. F. (2001b). Transfer of *Rhodopseudomonas acidophila* to the new genus *Rhodoblastus* as *Rhodoblastus acidophilus* gen. nov., comb. nov. *Int. J. Syst. Evol. Microbiol.* 51**,** 1863-1866. doi: 10.1099/00207713-51-5-1863.

Imhoff, J. F. (2003). Phylogenetic taxonomy of the family *Chlorobiaceae* on the basis of 16S rRNA and *fmo* (Fenna-Matthews-Olson protein) gene sequences. *Int. J. Syst. Evol. Microbiol.* 53**,** 941-951. doi: 10.1099/ijs.0.02403-0.

Imhoff, J. F. (2014). "The family Chlorobiaceae," in *The prokaryotes,* eds. E. Rosenberg, E.F. DeLong, S. Lory, E. Stackebrandt & F. Thompson. (Berlin Heidelberg: Springer), 501-514.

Imhoff, J. F. (2015a). "*Rhodopseudomonas,*" in *Bergey's manual of systematics of Archaea and Bacteria*. (: John Wiley & Sons, Inc. in association with Bergey's Manual Trust), 1-13.

Imhoff, J. F. (2015b). "*Rhodospirillum,*" in *Bergey's manual of systematics of Archaea and Bacteria*. John Wiley & Sons, Inc. in association with Bergey's Manual Trust), 1-10.

Imhoff, J. F., and Pfennig, N. (2001). *Thioflavicoccus mobilis* gen. nov., sp nov., a novel purple sulfur bacterium with bacteriochlorophyll *b*. *Int. J. Syst. Evol. Microbiol.* 51**,** 105-110. doi: 10.1099/00207713-51-1-105.

Imhoff, J. F., Süling, J., and Petri, R. (1998). Phylogenetic relationships among the *Chromatiaceae*, their taxonomic reclassification and description of the new genera *Allochromatium*, *Halochromatium*, *Isochromatium*, *Marichromatium*, *Thiococcus*, *Thiohalocapsa*, and *Thermochromatium Int. J. Syst. Bacteriol.* 48**,** 1129-1143. doi: 10.1099/00207713-48-4-1129.

Imhoff, J. F., and Thiel, V. (2010). Phylogeny and taxonomy of Chlorobiaceae. *Photosynth. Res.* 104**,** 123-136. doi: 10.1007/s11120-009-9510-7.

Ivanovsky, R. N., Keppen, O. I., Lebedeva, N. N., Beletsky, A. V., Mardanov, A. V., and Grouzdev, D. S. (2017). Draft genome sequence of the anoxygenic phototrophic bacterium *Phaeospirillum fulvum* MGU-K5. *Genome Announc.* 5**,** e00895-00817. doi: 10.1128/genomeA.00895-17.

Kelly, D. P. (2008). Stable sulfur isotope fractionation by the green bacterium *Chlorobaculum parvum* during photolithoautotrophic growth on sulfide. *Pol. J. Microbiol.* 57**,** 275-279.

Kojima, H., and Fukui, M. (2010). *Sulfuricella denitrificans* gen. nov., sp. nov., a sulfur-oxidizing autotroph isolated from a freshwater lake. *Int. J. Syst. Evol. Microbiol.* 60**,** 2862-2866. doi: 10.1099/ijs.0.016980-0.

Kojima, H., Ogura, Y., Yamamoto, N., Togashi, T., Mori, H., Watanabe, T., et al. (2015). Ecophysiology of *Thioploca ingrica* as revealed by the complete genome sequence supplemented with proteomic evidence. *ISME J.* 9**,** 1166-1176. doi: 10.1038/ismej.2014.209.

Kuwahara, H., Yoshida, T., Takaki, Y., Shimamura, S., Nishi, S., Harada, M., et al. (2007). Reduced genome of the thioautotrophic intracellular symbiont in a deep-sea clam, *Calyptogena okutanii*. *Current Biology* 17**,** 881-886. doi: 10.1016/j.cub.2007.04.039.

Lapidus, A., Nolan, M., Lucas, S., Glavina Del Rio, T., Tice, H., Cheng, J. F., et al. (2011). Genome sequence of the filamentous, gliding *Thiothrix nivea* neotype strain (JP2^T). *Stand. Genomic Sci.* 5**,** 398-406. doi: 10.4056/sigs.2344929.

Madigan, M. T. (1986). *Chromatium tepidum* sp. nov., a thermophilic photosynthetic bacterium of the family Chromatiaceae. *Int. J. Syst. Bacteriol.* 36**,** 222-227. doi: 10.1099/00207713-36-2-222.

Mansor, M., Hamilton, T. L., Fantle, M. S., and Macalady, J. L. (2015). Metabolic diversity and ecological niches of *Achromatium* populations revealed with single-cell genomic sequencing. *Front. Microbiol.* 6**,** 822. doi: 10.3389/fmicb.2015.00822.

Mansor, M., and Macalady, J. L. (2016). Draft genome sequence of Lampenflora *Chlorobium limicola* strain Frasassi in a sulfidic cave system. *Genome Announc.* 4**,** e00357-00316. doi: 10.1128/genomeA.00357-16.

Matsunaga, T., Okamura, Y., Fukuda, Y., Wahyudi, A. T., Murase, Y., and Takeyama, H. (2005). Complete genome sequence of the facultative anaerobic magnetotactic bacterium *Magnetospirillum* sp. strain AMB-1. *DNA Res.* 12**,** 157-166. doi: 10.1093/dnares/dsi002.

Miroshnichenko, M. L., Gongadze, G. A., Lysenko, A. M., and Bonch-Osmolovskaya, E. A. (1994). *Desulfurella multipotens* sp. nov., a new sulfur-respiring thermophilic eubacterium from Raoul Island (Kermadec archipelago, New Zealand). *Arch. Microbiol.* 161**,** 88-93. doi: 10.1007/bf00248898.

Momper, L., Jungbluth, S. P., Lee, M. D., and Amend, J. P. (2017). Energy and carbon metabolisms in a deep terrestrial subsurface fluid microbial community. *ISME J.* 11**,** 2319-2333. doi: 10.1038/ismej.2017.94.

Moran, M. A., Gonzalez, J. M., and Kiene, R. P. (2003). Linking a bacterial taxon to sulfur cycling in the sea: Studies of the marine *Roseobacter* group. *Geomicrobiol. J.* 20**,** 375-388. doi: 10.1080/01490450303901.

Mussmann, M., Richter, M., Lombardot, T., Meyerdierks, A., Kuever, J., Kube, M., et al. (2005). Clustered genes related to sulfate respiration in uncultured prokaryotes support the theory of their concomitant horizontal transfer. *J. Bacteriol.* 187**,** 7126-7137. doi: 10.1128/JB.187.20.7126-7137.2005.

Newton, I. L. G., Woyke, T., Auchtung, T. A., Dilly, G. F., Dutton, R. J., Fisher, M. C., et al. (2007). The *Calyptogena magnifica* chemoautotrophic symbiont genome. *Science* 315**,** 998-1000. doi: 10.1126/science.1138438.

Nunoura, T., Takaki, Y., Kazama, H., Kakuta, J., Shimamura, S., Makita, H., et al. (2014). Physiological and genomic features of a novel sulfur-oxidizing Gammaproteobacterium belonging to a previously uncultivated symbiotic lineage isolated from a hydrothermal vent. *PLoS ONE* 9**,** e104959. doi: 10.1371/journal.pone.0104959.

Orlygsson, J., and Kristjansson, J. K. (2014). "The family *Hydrogenophilaceae*," in *The prokaryotes - Alphaproteobacteria and Betaproteobacteria*, eds. E. Rosenberg, E.F. DeLong, S. Lory, E. Stackebrandt & F. Thompson. (Berlin Heidelberg: Springer-Verlag), 859-868.

Overmann, J., and Pfennig, N. (1989). *Pelodictyon phaeoclathratiforme* sp. nov., a new brown-colored member of the Chlorobiaceae forming net-like colonies. *Arch. Microbiol.* 152**,** 401-406. doi: 10.1007/bf00425181.

Parks, D. H., Chuvochina, M., Waite, D. W., Rinke, C., Skarshewski, A., Chaumeil, P.-A., et al. (2018). A standardized bacterial taxonomy based on genome phylogeny substantially revises the tree of life. *Nat. Biotechnol.* 36**,** 996-1004. doi: 10.1038/nbt.4229.

Petersen, J. M., Zielinski, F. U., Pape, T., Seifert, R., Moraru, C., Amann, R., et al. (2011). Hydrogen is an energy source for hydrothermal vent symbioses. *Nature* 476**,** 176-180. doi: 10.1038/nature10325.

Probst, A. J., Castelle, C. J., Singh, A., Brown, C. T., Anantharaman, K., Sharon, I., et al. (2017). Genomic resolution of a cold subsurface aquifer community provides metabolic insights for novel microbes adapted to high CO_2 concentrations. *Environ. Microbiol.* 19**,** 459-474. doi: 10.1111/1462-2920.13362.

Probst, A. J., Elling, F. J., Castelle, C. J., Zhu, Q., Elvert, M., Birarda, G., et al. (2020). Lipid analysis of CO_2-rich subsurface aquifers suggests an autotrophy-based deep biosphere with lysolipids enriched in CPR bacteria. *ISME J.* 14**,** 1547-1560. doi: 10.1038/s41396-020-0624-4.

Probst, A. J., Ladd, B., Jarett, J. K., Geller-McGrath, D. E., Sieber, C. M. K., Emerson, J. B., et al. (2018). Differential depth distribution of microbial function and putative symbionts through sediment-hosted aquifers in the deep terrestrial subsurface. *Nat. Microbiol.* 3**,** 328-336. doi: 10.1038/s41564-017-0098-y.

Rasigraf, O., van Helmond, N. A. G. M., Frank, J., Lenstra, W. K., Egger, M., Slomp, C. P., et al. (2019). Metagenomic analysis reveals large potential for carbon, nitrogen and sulfur cycling in coastal methanic sediments of the Bothnian Sea. *bioRxiv* preprint. doi: 10.1101/553131.

Russell, S. L., Corbett-Detig, R. B., and Cavanaugh, C. M. (2017). Mixed transmission modes and dynamic genome evolution in an obligate animal-bacterial symbiosis. *ISME J.* 11**,** 1359-1371. doi: 10.1038/ismej.2017.10.

Smith, J. A. (2014). *A novel manganese oxidising bacterium: characterisation and genomic evaluation.* PhD, University of Auckland.

Sorokin, D. Y., Muntyan, M. S., Panteleeva, A. N., and Muyzer, G. (2012). *Thioalkalivibrio sulfidiphilus* sp. nov., a haloalkaliphilic, sulfur-oxidizing gammaproteobacterium from alkaline habitats. *Int. J. Syst. Evol. Microbiol.* 62**,** 1884-1889. doi: 10.1099/ijs.0.034504-0.

Sorokin, D. Y., Tourova, T. P., Lysenko, A. M., Mityushina, L. L., and Kuenen, J. G. (2002). *Thioalkalivibrio thiocyanoxidans* sp. nov. and *Thioalkalivibrio paradoxus* sp. nov., novel alkaliphilic, obligately autotrophic, sulfur-oxidizing bacteria capable of growth on thiocyanate, from soda lakes. *Int. J. Syst. Evol. Microbiol.* 52**,** 657-664. doi: 10.1099/00207713-52-2-657.

Stamps, B. W., Leddy, M. B., Plumlee, M. H., Hasan, N. A., Colwell, R. R., and Spear, J. R. (2018). Characterization of the microbiome at the world's largest potable water reuse facility. *Front. Microbiol.* 9**,** 2435. doi: 10.3389/fmicb.2018.02435.

Tan, S., Liu, J., Fang, Y., Hedlund, B. P., Lian, Z. H., Huang, L. Y., et al. (2019). Insights into ecological role of a new deltaproteobacterial order *Candidatus* Acidulodesulfobacterales by metagenomics and metatranscriptomics. *ISME J.* 13**,** 2044–2057. doi: 10.1038/s41396-019-0415-y.

Tank, M., Liu, Z., Frigaard, N. U., Tomsho, L. P., Schuster, S. C., and Bryant, D. A. (2017). Complete genome sequence of the photoautotrophic and bacteriochlorophyll e-synthesizing green sulfur bacterium *Chlorobaculum limnaeum* DSM 1677^{T}. *Genome Announc.* 5**,** e00529-00517. doi: 10.1128/genomeA.00529-17.

Thiel, V., Garcia Costas, A. M., Fortney, N. W., Martinez, J. N., Tank, M., Roden, E. E., et al. (2019). "*Candidatus* Thermonerobacter thiotrophicus," a non-phototrophic member of the Bacteroidetes/Chlorobi with dissimilatory sulfur metabolism in hot spring mat communities. *Front. Microbiol.* 9**,** 3159. doi: 10.3389/fmicb.2018.03159.

Todd, J. D., Kirkwood, M., Newton-Payne, S., and Johnston, A. W. (2012). DddW, a third DMSP lyase in a model Roseobacter marine bacterium, *Ruegeria pomeroyi* DSS-3. *ISME J.* 6**,** 223-226. doi: 10.1038/ismej.2011.79.

Trubitsyn, D., Abreu, F., Ward, F. B., Taylor, T., Hattori, M., Kondo, S., et al. (2016). Draft genome sequence of *Magnetovibrio blakemorei* strain MV-1, a marine vibrioid magnetotactic bacterium. *Genome Announc.* 4**,** e01330-01316. doi: 10.1128/genomeA.01330-16.

Tully, B. J., Graham, E. D., and Heidelberg, J. F. (2018). The reconstruction of 2,631 draft metagenome-assembled genomes from the global oceans. *Sci. Data* 5**,** 170203. doi: 10.1038/sdata.2017.203.

Vogl, K., Glaeser, J., Pfannes, K. R., Wanner, G., and Overmann, R. (2006). *Chlorobium chlorochromatii* sp. nov., a symbiotic green sulfur bacterium isolated from the phototrophic consortium " Chlorochromatium aggregatum ". *Arch. Microbiol.* 185**,** 363-372. doi: 10.1007/s00203-006-0102-z.

Wang, X., Wang, Q., Zhang, W., Wang, Y., Li, L., Wen, T., et al. (2014). Complete genome sequence of *Magnetospirillum gryphiswaldense* MSR-1. *Genome Announc.* 2**,** e00171-00114. doi: 10.1128/genomeA.00171-14.

Watanabe, T., Kojima, H., and Fukui, M. (2014). Complete genomes of freshwater sulfur oxidizers *Sulfuricella denitrificans* skB26 and *Sulfuritalea hydrogenivorans* sk43H: genetic insights into the sulfur oxidation pathway of betaproteobacteria. *Syst. Appl. Microbiol.* 37**,** 387-395. doi: 10.1016/j.syapm.2014.05.010.

Watanabe, T., Kojima, H., Shinohara, A., and Fukui, M. (2016). *Sulfurirhabdus autotrophica* gen. nov., sp. nov., isolated from a freshwater lake. *Int. J. Syst. Evol. Microbiol.* 66**,** 113-117. doi: 10.1099/ijsem.0.000679.

Watanabe, T., Kojima, H., Umezawa, K., Hori, C., Takasuka, T. E., Kato, Y., et al. (2019). Genomes of neutrophilic sulfur-oxidizing chemolithoautotrophs representing 9 proteobacterial species from 8 genera. *Front. Microbiol.* 10**,** 316. doi: 10.3389/fmicb.2019.00316.

Waterworth, S. C., Isemonger, E. W., Rees, E. R., Dorrington, R. A., and Kwan, J. C. (2020). Conserved bacterial genomes from two geographically distinct peritidal stromatolite formations shed light on potential functional guilds. *bioRxiv*. doi: 10.1101/818625.

Weissgerber, T., Zigann, R., Bruce, D., Chang, Y. J., Detter, J. C., Han, C., et al. (2011). Complete genome sequence of *Allochromatium vinosum* DSM 180^{T}. *Stand. Genomic Sci.* 5**,** 311-330. doi: 10.4056/sigs.2335270.

Zaar, A., Fuchs, G., Golecki, J. R., and Overmann, J. (2003). A new purple sulfur bacterium isolated from a littoral microbial mat, *Thiorhodococcus drewsii* sp. nov. *Arch. Microbiol.* 179**,** 174-183. doi: 10.1007/s00203-002-0514-3.

Zhang, G., Haroon, M. F., Zhang, R., Dong, X., Wang, D., Liu, Y., et al. (2017). *Ruegeria profundi* sp. nov. and *Ruegeria marisrubri* sp. nov., isolated from the brine-seawater interface at Erba Deep in the Red Sea. *Int. J. Syst. Evol. Microbiol.* 67**,** 4624-4631. doi: 10.1099/ijsem.0.002344.

Zhou, Z., Liu, Y., Xu, W., Pan, J., Luo, Z. H., and Li, M. (2020). Genome- and community-level interaction insights into carbon utilization and element cycling functions of Hydrothermarchaeota in hydrothermal sediment. *mSystems* 5**,** e00795-00719. doi: 10.1128/mSystems.00795-19.
